# Extending *N*-heterocyclic carbene ligands into the third dimension: a new type of hybrid phosphazane/NHC system† ‡
†Electronic supplementary information (ESI) available: Methods and additional data. CCDC 1040123 1040125–1040128. For ESI and crystallographic data in CIF or other electronic format see DOI: 10.1039/c4sc03966a

‡Dedicated to Professor Manfred Scheer on the occasion of his 60th birthday.


**DOI:** 10.1039/c4sc03966a

**Published:** 2015-02-13

**Authors:** Torsten Roth, Vladislav Vasilenko, Callum G. M. Benson, Hubert Wadepohl, Dominic S. Wright, Lutz H. Gade

**Affiliations:** a Anorganisch-Chemisches Institut , Universität Heidelberg , Im Neuenheimer Feld 270 , 69120 Heidelberg , Germany . Email: lutz.gade@uni-heidelberg.de; b Chemistry Department , Cambridge University , Lensfield Road , Cambridge , CB2 1EW , UK . Email: dsw1000@cam.ac.uk

## Abstract

A simple, “click” synthetic approach to a new type of hybrid phosph(III)azane/NHC system is described. The presence of the phosphazane P_2_N_2_ ring unit, with P atoms flanking the *NCN* fragment and with this ring perpendicular to the binding site of the NHC, provides unique opportunities for modifying the electronic and steric character of these carbenes.

## Abstract 2

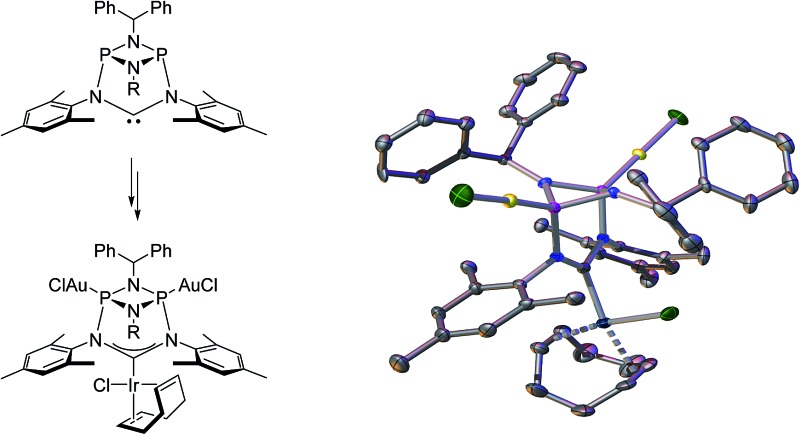
A new type of hybrid phosph(III)azane/NHC system is described in which a phosphazane P_2_N_2_ ring provides unique opportunities for modifying the electronic and steric character of these carbenes.

## Introduction

**Fig. 1:**
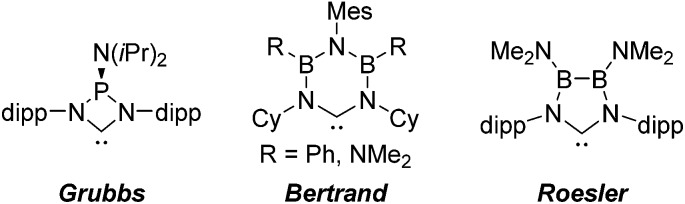
Selection of “inorganic” carbenes.

*N*-heterocyclic carbenes have been successfully established as versatile ligands in coordination chemistry, as powerful ancillary ligands in catalysis, as organocatalysts, and for an increasing number of other applications.^1–6^ In view of these applications the ability to vary the steric and electronic character of these ligands systematically, using simple modular synthetic routes, is a central theme in this area. In recent years a range of approaches to modify their σ-donor/π-acceptor and coordination properties have been implemented,^7^ for instance different ring sizes of the backbone resulting in different *NCN*-angles,^8^ variation of the heteroatoms adjacent to carbon,^9^ abnormal and remote carbenes,^10^ acyclic carbenes,^11^ anti-Bredt NHCs,^12^ push–pull carbenes,^13^ redox-switchable NHCs^14^ or even cyclophane-derived carbenes.^15^ A less explored approach relies on NHCs comprising “inorganic” or organometallic fragments in their ligand backbones (relevant examples shown in Fig. 1).^16^ This is, however, potentially a very powerful approach to ligand modification since introducing flanking electron-donating (*e.g.*, P) or electron-accepting (*e.g.*, B) main group atoms should have a profound effect on the acceptor or donor ability of the *NCN*-unit.

In contrast, much less effort has been dedicated to the systematic development of NHC ligands with unusual and improved spatial characteristics.^17^ Most approaches focussed on the introduction of various bulky substituents in the *wingtip* position. Although a variety of backbone structures containing heteroatoms have been reported in recent years NHCs are still regarded as essentially two-dimensional ancillary ligands. Even though NHCs have proven to be superior to phosphine ligands in several respects, the latter are often chosen preferentially due to their three-dimensional shape, which is of relevance *inter alia* in enantioselective homogenous catalysis.^18^

**Fig. 2:**
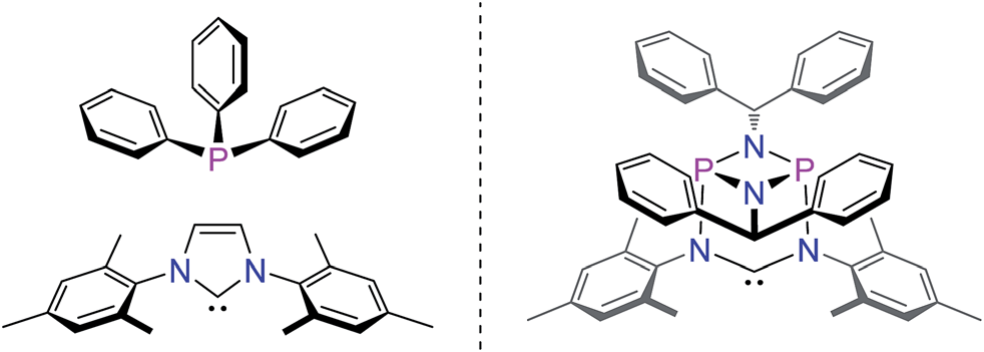
Spatial characteristics of phosphines, NHCs (left) and the targeted 3-dimensional hybrid phosphazane/NHC ligand (right).

Herein, we present a conceptually new approach for the design of the steric properties of NHC-ligands, which have so far only been perceived as essentially flat donor entities. Our system constitutes the first example of a three-dimensional arrangement encompassing the central *NCN*-unit that allows for straightforward control of the steric properties of the top and bottom hemispheres of the widespread NHC-ligand framework. This is achieved by a unique inorganic backbone moiety consisting of a cyclophosphazane P_2_N_2_-unit that is orthogonally attached to the *NCN*-fragment (Fig. 2).

Stemming from our interest in the modular synthesis of cyclophosph(III)azane ligands for catalysis,^19^ we have begun to explore the possibility of developing a simple “click-type” approach to ambidentate^20^ hybrid phosphazane/NHC ligands as shown in Fig. 2. We reasoned that the presence of the P_2_N_2_ ring unit (perpendicular and adjacent to the *NCN*-fragment) would provide a unique strategy for modifying the electronic and spatial characteristics of the NHC system.

## Results

**Fig. 3:**
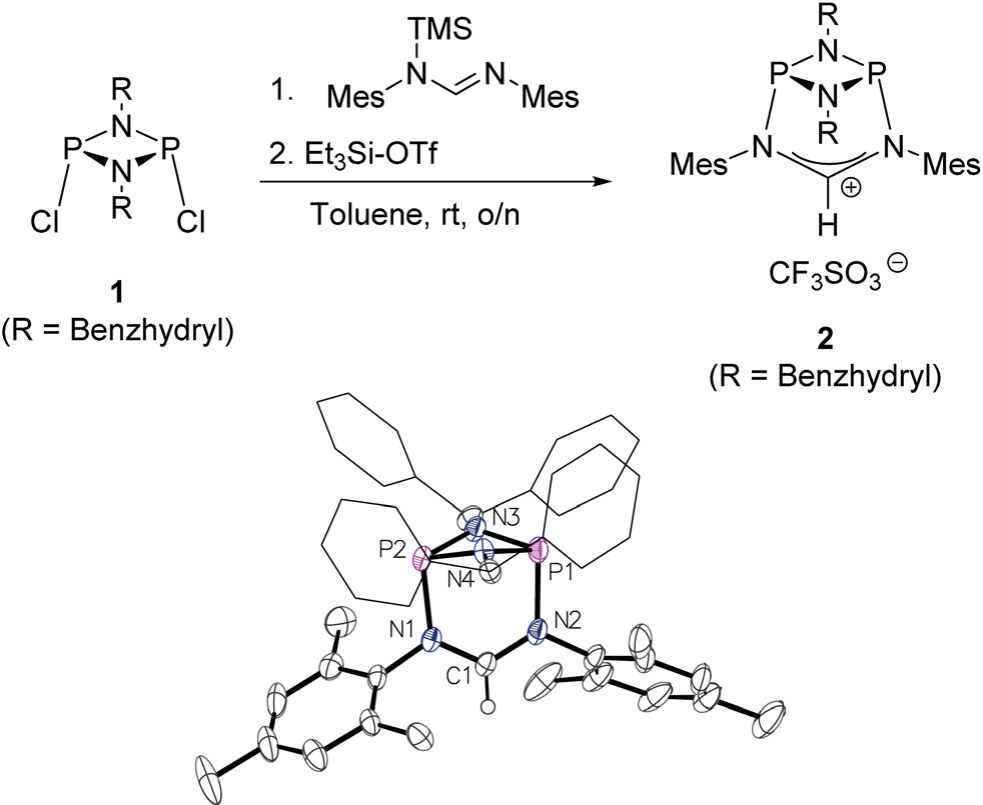
Top: one-pot access to the cationic cyclophosphazane cage compound **2**. Bottom: molecular structure of **2**, thermal ellipsoids set at the 50% probability level. H-atoms except H(1) and the trifluoromethanesulfonate counterion have been omitted for clarity. Phenyl rings of the benzhydryl substituents are drawn as wireframes. Selected bond lengths [Å] and angles [°]: P1–N2 1.837(3), P1–N3 1.704(3), N1–C1 1.323(4), N2–C1–N1 122.7(3).

The preparation of the new ligand system starts from simple Me_3_Si-protected diarylformamidines and dichloro-cyclophosphazanes, both of which are readily accessible on a multi-gram scale. Condensation of the two components and subsequent ring-closure using Et_3_Si-OTf furnishes cationic azolium salts,^21^ a key example of which is **2** (Fig. 3). The solid state structure of **2** confirms the bicyclic cage structure in which the *NCN*-unit bridges the P_2_N_2_-ring. The P_2_N_2_ moiety and its substituents are orthogonal to the carbene plane, thus effectively shrouding the posterior of the carbene system.

**Scheme 1:**
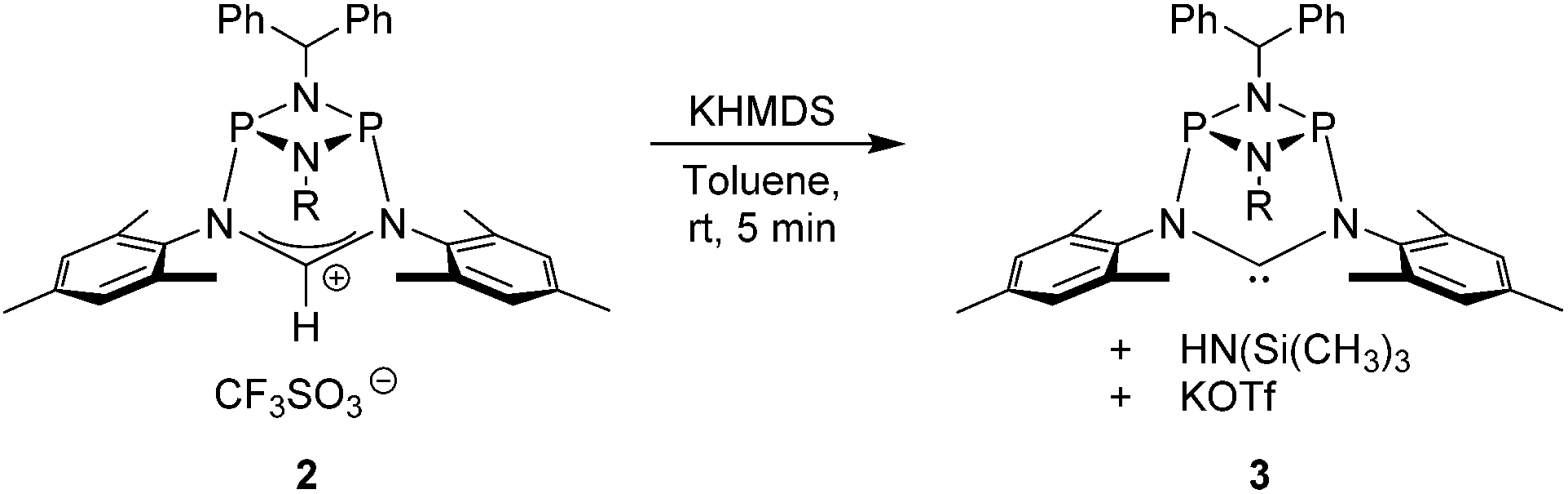
*In situ* generation of carbene **4**.

Notably, quantitative formation of carbene species **3** was observed upon addition of KHMDS to **2**, exhibiting a single ^31^P NMR signal (197 ppm) and a characteristic ^13^C NMR resonance at 260 ppm, corresponding to its carbene carbon atom, in the *in situ* recorded spectrum (Scheme 1). Although carbene species **3** is stable in solution for several hours, attempts to isolate the free carbene invariably led to partial decomposition.

**Scheme 2:**
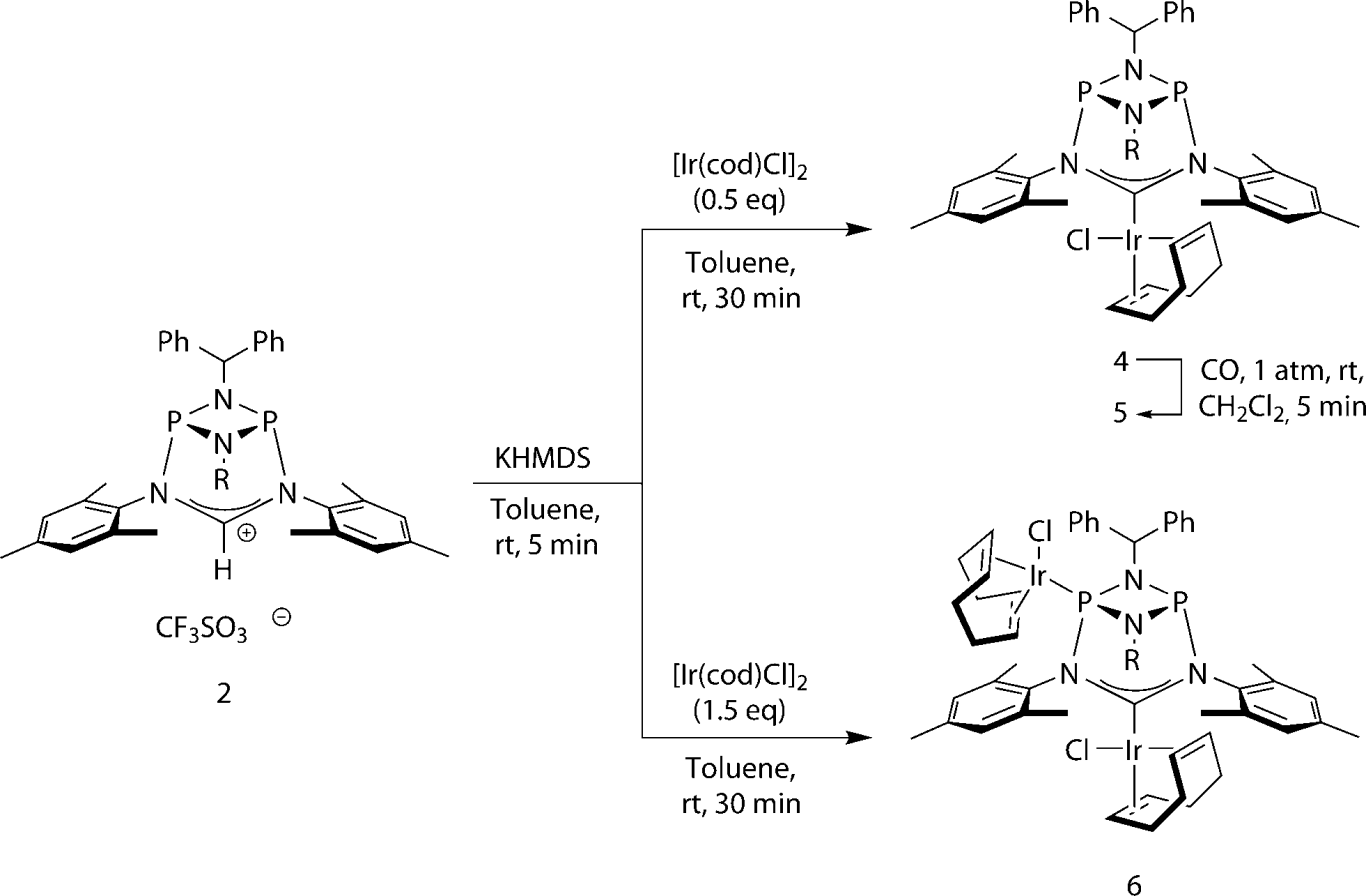
Metalation of carbene **3**.

**Fig. 4:**
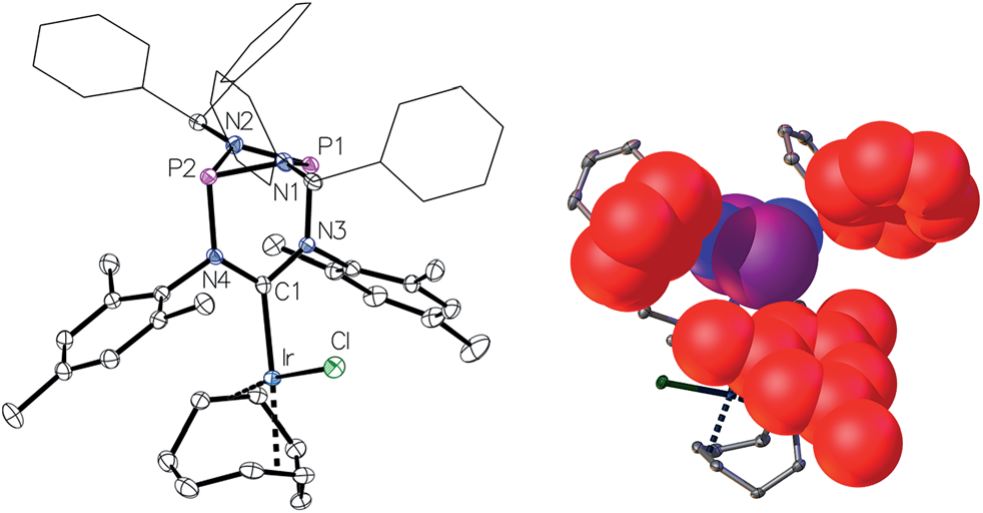
Left: molecular structure of **4**, thermal ellipsoids set at the 50% probability level. H-atoms have been omitted for clarity. Phenyl rings of the benzhydryl substituents are drawn as wireframes. Selected bond lengths [Å] and angles [°]: Ir–C1 2.040(2), P1–N3 1.7658(19), N3–C1 1.372(3), N3–C1–N4 114.06(19). Right: space-filling representation of one of the *C*_3_-symmetric binding pockets. Phenyl and mesityl rings marking the boundary of the cavity are highlighted in red.

We then turned to probing the reactivity of carbene **3** employing Lewis-acidic metal-fragments. Deprotonation of **2** and subsequent addition of half an equivalent of [Ir(cod)Cl]_2_ led to quantitative formation of the robust^22^ NHC–metal complex **4** (Scheme 2, Fig. 4, left). To classify the electron-donor properties of the carbene unit we determined the electrochemical characteristics of **4** and the TEP (*Tolman Electronic Parameter*)^23^ of the [Ir(CO)_2_Cl] carbene complex **5** (for the solid-state structure of **5** see ESI†).

The IR spectrum of **5** displays CO stretching vibrations at 1976 cm^–1^ and 2060 cm^–1^ with a calculated^24^ TEP value of 2045 cm^–1^ representing a two-electron donor capacity comparable to other six-membered “inorganic” carbenes (Fig. 1, Bertrand *et al.*).^16*c*^ Moreover, the cyclic voltammogram of **4** features a single quasi-reversible oxidation step (*E*_1/2_ = +0.653 V, Δ*E* = 159 mV),^25^ suggesting that the cyclophosphazane carbene is a powerful electron donor ligand. The strong σ-donor character of **3** is consistent with the presence of the donor-P atoms adjacent to the *NCN*-unit.

One of the most notable features of the solid-state structures of the iridium complexes **4** and **5** is the presence of two conical binding pockets of local *C*_3_ symmetry around the uncoordinated phosphorus atoms, the borders of these cavities being defined by the benzhydryl phenyl and mesityl rings (Fig. 4, right).

**Fig. 5:**
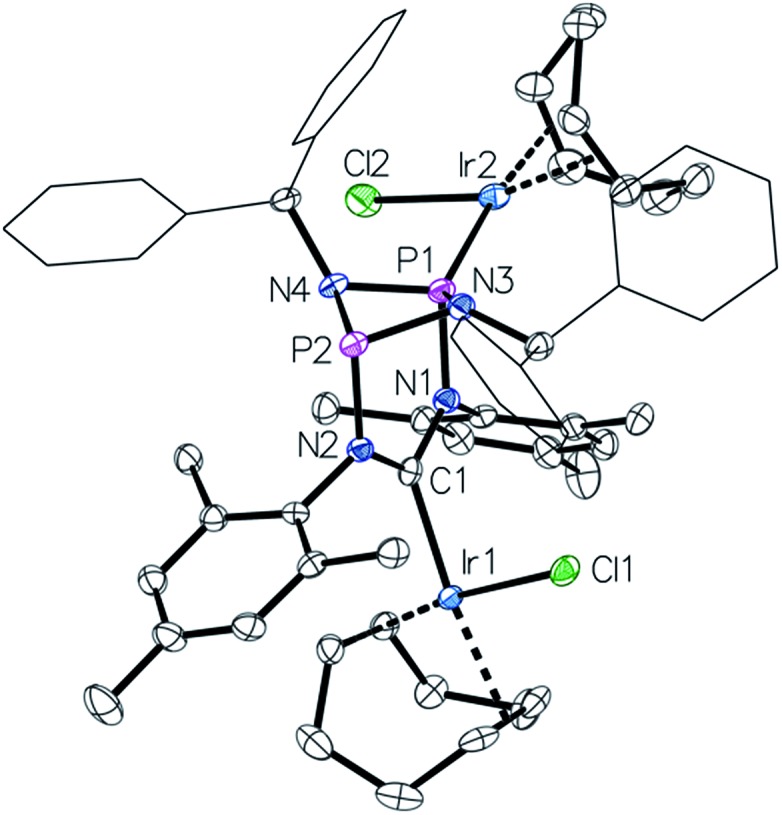
Molecular structure of **6**, thermal ellipsoids set at the 50% probability level. H-atoms have been omitted for clarity. Phenyl rings of the benzhydryl substituents are drawn as wireframes. Selected bond lengths [Å] and angles [°]: Ir1–C1 2.046(3), Ir2–P1 2.2359(8), P1–N1 1.756(3), N1–C1 1.385(4), Ir2–P1–P2 158.50(4), N2–C1–N1 114.9(3).

This led us to repeat the complexation reaction used in the formation of **4** employing 1.5 equivalents [Ir(cod)Cl]_2_. Surprisingly, the ^31^P NMR spectrum showed two doublets (*J*_P–P_ = 30 Hz), suggesting a dimetallic complex in which there is unsymmetrical coordination of the P_2_N_2_ ring. Even after the addition of an excess of the iridium precursor no further species were detected by ^31^P NMR, indicating that the lone pair of the second phosphorus atom is not accessible for coordination. The solid-state structure of the product **6** reveals that the coordination of the second iridium fragment by one phosphorus lone pair induces a slight internal rearrangement within the core structure, thus effectively sterically-blocking the second phosphorus coordination site (Fig. 5). Since **6** could only be isolated in pure form in the solid state and ^31^P NMR spectra showed a mixture of **4** and **6** in solution, a VT NMR study was conducted to probe the coordination behavior of the P_2_N_2_ phosphorus atoms. Crystals of complex **6** were dissolved in THF and the resulting equilibrium was studied in the temperature range –30 to 50 °C. A labile P–Ir bond and an entropically driven reversible dissociation process was found (**6** → **4** + 1/2[Ir(cod)Cl]_2_, Δ*H* = +30 kJ mol^–1^, Δ*S* = +77 J mol^–1^).

**Fig. 6:**
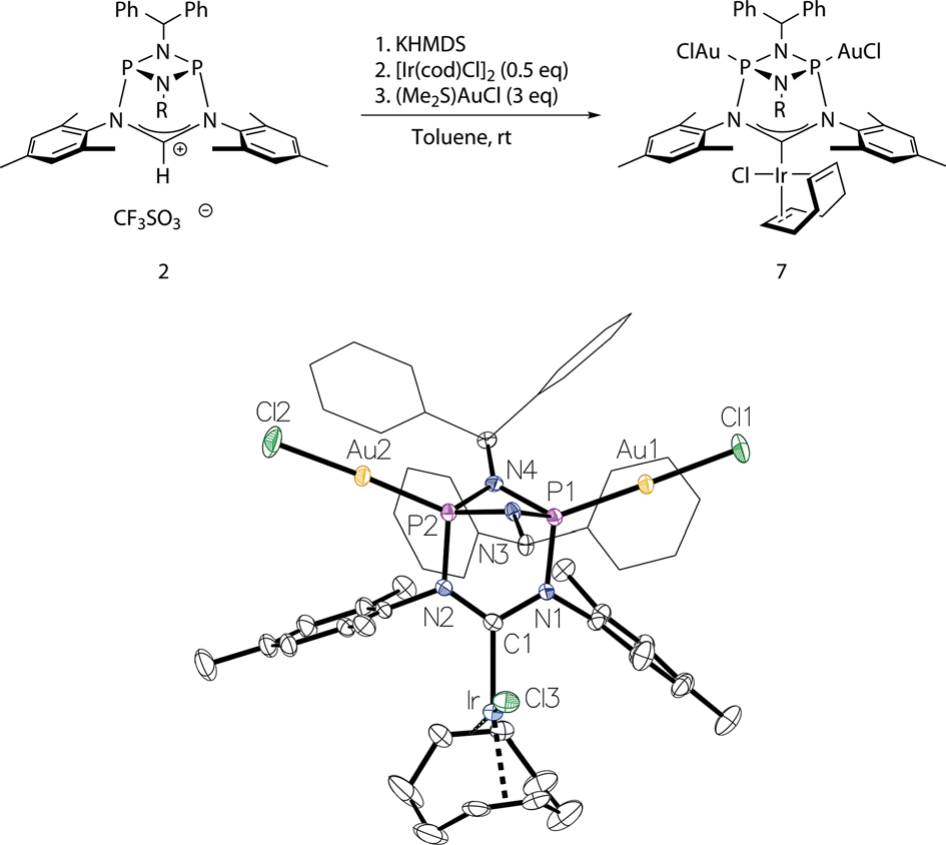
Top: access to the trimetallic complex **7**. Bottom: molecular structure of **7**, thermal ellipsoids set at the 50% probability level. H-atoms have been omitted for clarity. Phenyl rings of the benzhydryl substituents are drawn as wireframes. Selected bond lengths [Å] and angles [°]: Au1–P1 2.1839(10), Ir–C1 2.020(4), P1–N1 1.719(4), P1–N3 1.712(4), N1–C1 1.390(5), N2–C1–N1 115.3(4).

Moreover, to preclude electronic effects as the cause for the bismetalation product **6**, we exchanged the square-planar [Ir]-precursor for a sterically less demanding linear gold(i) fragment. By a procedure analogous to the preparation of **6** the heterotrimetallic complex **7** was obtained (Fig. 6). Consequently, the ^13^C NMR shift of the carbene carbon atom of *δ* = 234 ppm (t, *J*_C–P_ = 4.5 Hz) corroborates coordination of the [Ir(cod)Cl] fragment and the single ^31^P NMR resonance of 128 ppm establishes metalation of both phosphorus atoms. Details of the molecular structure have also been established by X-ray diffraction (Fig. 6). Complex **7** was also converted to its corresponding carbonyl complex by the previously mentioned procedure for compound **5**. However, significant decomposition precluded its isolation in a pure state. The carbonyl complex exhibits a ^31^P NMR resonance at 131 ppm and the IR spectrum shows two CO stretching vibrations at 2071 cm^–1^ and 1986 cm^–1^ (*ν*_av_(CO) = 2029 cm^–1^; TEP = 2054 cm^–1^).

**Fig. 7:**
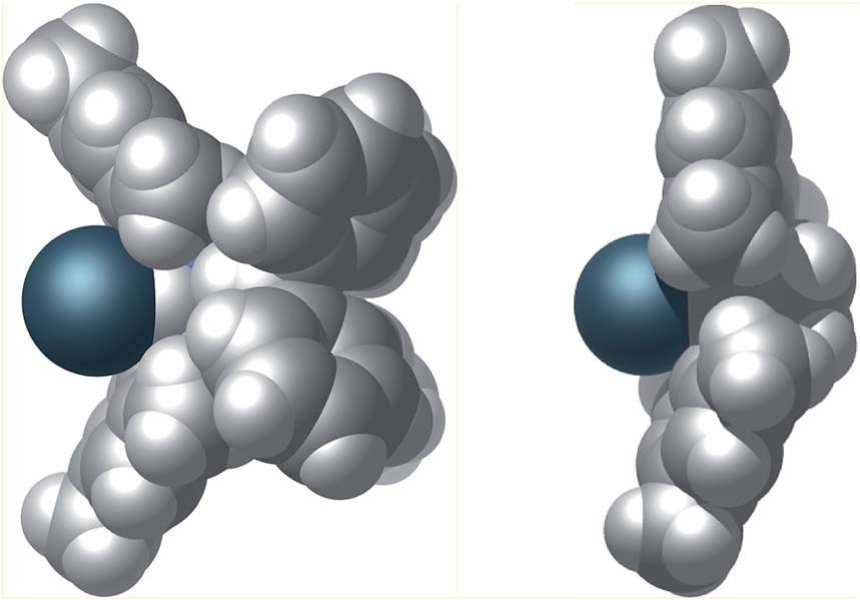
CPK model of the van der Waals surface of complex **4** (left) and, for comparison complex SIMes·[Ir(cod)Cl] (right).^26^ Cyclooctadiene and chlorido ligands have been omitted for clarity.

Finally, the steric properties of the ligand system were analyzed. The extended heteroatomic backbone leads to a more confined metal coordination site compared to the standard SIMes NHC (Fig. 7). This effect is accomplished by a smaller angle between the mesityl substituents and the presence of the bulky P_2_N_2_ substituents. Moreover the CPK model demonstrates the close spatial proximity of the benzhydryl phenyl groups to the iridium binding pocket, thus effectively shielding the backside of the NHC.

To obtain a quantitative measure for the steric congestion of the metal centre we have determined buried volumes %*V*_bur_ of all isolated complexes for the NHC and the phosphorus donor sites (Table 1).^27^ As expected, all phosphazane–NHC complexes reported in this work feature increased buried volumes compared to the SIMes reference system. Remarkably, the steric properties of the NHC–metal unit are unaffected by the coordination of metal fragments to the P_2_N_2_ backbone. This offers a convenient way to modify the NHC donor strength independently from the sterics.

**Table 1:** Calculated buried volume %*V*_bur_ of the reported P_2_N_2_ cages^27^

Compound	%*V*_bur_-NHC	%*V*_bur_-P_(1)_	%*V*_bur_-P_(2)_
**2**	45.6	55.3	52.9
**4**	36.7	52.7	57.7
**5**	38.8	56.9	49.9
**6**	37.3	41.9^*a*^	60.6
**7**	36.7	55.4	57.6

^*a*^Phosphorus atom is coordinated to [Ir(cod)Cl].

## Conclusion

We have established a cyclic P_2_N_2_ unit as a stable “inorganic” carbene backbone moiety within a new type of polytopical hybrid phosph(III)azane/NHC system. The new ligand is structurally rigid and a strong σ-donor. Importantly, the essentially flat spatial arrangement of conventional NHCs, often described as fan-, wedge- or fence-like, is extended into the third dimension in our new system, in which the orthogonal disposition of the P_2_N_2_ ring unit provides a unique architecture for tuning the steric and spatial properties of the carbene binding site. The ability of these new ligands to coordinate metal centres using their P-atoms provides a further means by which the steric and electronic character of the carbene fragment can be modified. In addition, the simple “click” approach may allow a broad range of hybrid NHCs of this type to be prepared, with (for example) the future potential for readily introduced chiral functionality at the phosphazane-N atom. Further studies in this area are ongoing.
