# Supplementary material for: Extending N-heterocyclic carbene ligands into the third dimension: a new type of hybrid phosphazane/NHC system
Source: Chem Sci. 2015 Feb 13;6(4):2506–10. doi: 10.1039/c4sc03966a (PMC5647813; doi:10.1039/c4sc03966a)
Supplement: Supplementary file 1 [file SC-006-C4SC03966A-s001.pdf]

This journal is © The Royal Society of Chemistry 2015

Contents:

1. General Information……………………………………………………………………..………S-2

2. Preparation of the starting materials……………………………………...……...………S-3

3. Preparation and Characterization of **2**, **3**, **4**, **5**, **6**, **7**….…………………………………………S-5

4. Variable Temperature Experiments……………………………...…………………..……S-13

6. X-ray Crystal Structure Determinations……………………………………………...…S-15

7. Solid State Structures…………………………………..……………………….…..…S-17

8. Space-filling representations of the donor sites………………..……………….……..S-22

9. Selected NMR Spectra………..…………………..…………………..………………………S-23

10. References………………………………………………………………………….……S-32

1. General Information

All manipulations, except those indicated, were carried out under exclusion of air and moisture using standard Schlenk and glove box techniques. As inert gas, Argon 5.0, purchased from Messer Group GmbH, was used after drying over Granusic© phosphorpentoxide granulate. Solvents were dried over activated alumina columns using a solvent purification system (M. Braun SPS 800) or according to standard literature-known methods[1] and stored in glass ampules under an argon atmosphere. Diethyl ether and *n*-pentane were distilled from sodium/potassium alloy, tetrahydrofuran, benzene and *n*-hexane from potassium, methanol from magnesium, dichloromethane, chloroform and triethylamine from calcium hydride and toluene from sodium. The same procedures were used to dry the deuterated solvents. Degassed solvents were obtained by three successive *freeze-pump-thaw*-cycles. Phosphorus trichloride was distilled prior to use and triethylamine was degassed. NMR spectra were recorded on Bruker Avance (400 MHz, 500 MHz, 600 MHz) instruments. Chemical shifts (δ) are reported in parts per million (ppm) and are referenced to residual proton solvent signals or carbon resonances.[2] H_3PO_4 (^{31}P) and CCl_3F (^{19}F) were used as external standards. The following abbreviations were used: s (singlet), d (doublet), dd (doublet of doublets), t (triplet), m (multiplet), br (broad signal). High-resolution mass spectra were acquired on Bruker ApexQe hybrid 9.4 T FT-ICR (ESI) and JEOL JMS-700 magnetic sector (FAB, EI, LIFDI) spectrometers at the mass spectrometry facility of the Institute of Organic Chemistry, of the University of Heidelberg. Elemental analyses were carried out in the Microanalysis Laboratory of the Heidelberg Chemistry Department on a vario MICRO cube (Elementar*)*. IR spectra were acquired on a Varian 3100 FT IR spectrometer (Excalibur series) in a CH_2Cl_2 solution of the compounds at room temperature using a CaF_2 cell. All chemicals were obtained from commercial suppliers and were used without further purification.

2. Preparation of the starting materials

Benzhydryl-dichlorocyclophosphazane[3]

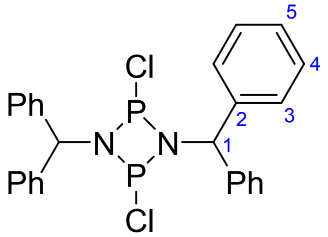

1

1[3] was synthesized from PCl_3 and benzhydrylamine according to a reported procedure.

^{1}H-NMR (thf-d_8, 399.89 MHz, 295 K): δ [ppm] = 7.58-7.49 (m, 8H, H-3), 7.33-7.26 (m, 8H, H-4), 7.25-7.19 (m, 4H, H-5), 5.70 (t, *J*= 6.15 Hz, 2H, H-1);

^{13}C{^{1}H}-NMR (thf-d_8, 100.56 MHz, 295 K): δ [ppm] = 139.89 (s, C-2), 129.39 (s, C-4), 128.92 (s, C-3), 128.73 (s, C-5), 63.95 (t, J= 6.82 Hz, C-1);

^{31}P{^{1}H}-NMR (thf-d_8, 161.89 MHz, 295 K): δ [ppm] = 223.86 (s);

**MS (LIFDI(+)):**	m / z	493.9	($[M]^+$)
	calculated:	494.1	($C_{26}H_{22}N_2P_2Cl_2$ ≙ $[M]^+$).

***N,N'*-Bis(2,4,6-trimethylphenyl)formamidine[4]**

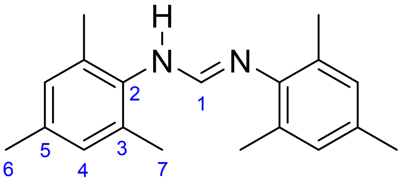

N,N'-Bis(2,4,6-trimethylphenyl)formamidine was prepared following a method described by Grubbs and Kuhn.[4] Two rotational isomers are present in C_6D_6 solution at room temperature in a *ca.* 1:1 ratio:

^{1}H-NMR (C_6D_6, 399.89 MHz, 296 K): <u>Isomer-1</u>: δ [ppm] = 6.98 (d, *J*= 11.8 Hz, 1H, H-1/N-*H*), 6.93 (s, 2H, H-4), 6.58 (s, 2H, H-4), 5.04 (d, *J*= 11.8 Hz, 1H, H-1/N-*H*), 2.35 (s, 6H, H-7), 2.26

(s, 3H, H-6), 2.06 (s, 3H, H-6’), 1.89 (s, 6H, H-7’); Isomer-2: δ [ppm] =6.87 (s, 1H, H-1 / N-H), 6.77 (s, 4H, H-4), 2.20 (s, 12H, H-7), 2.15 (s, 6H, H-6);

Spectral data match the reported.[4],[5]

**MS (HR-EI(+)):**	m / z	280.1935	$([M]^+)$
	calculated:	280.1939	$(C_{19}H_{24}N_2 \hat{=} [M]^+)$

***N*,*N’*-Bis(2,4,6-trimethylphenyl)-*N*-trimethylsilylformamidine**[6]

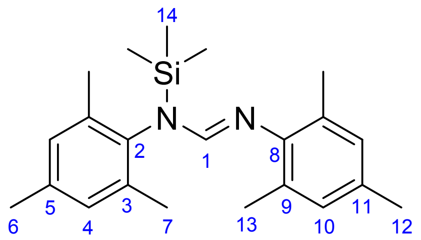

N,*N’*-Bis(2,4,6-trimethylphenyl)-*N*-trimethylsilylformamidine was prepared following a method described by Grubbs and Despagnet-Ayoub.[6]

^{1}H-NMR (C_6D_6, 600.13 MHz, 295 K): δ [ppm] = 6.89-6.67 (bs, 1H, H-1), 6.86 (s, 2H, H-4/10), 6.72 (s, 2H, H-4/10), 2.25 (s, 3H, H-6/12), 2.24 (s, 6H, H-7/13), 2.14 (s, 6H, H-7/13), 2.11 (s, 3H, H-6/12), 0.36 (s, 9H, H-14);

^{13}C{^{1}H}-NMR (C_6D_6, 150.90 MHz, 295 K): δ [ppm] = 156.79 (C-1), 148.33 (C_{Ar}), 139.81 (C_{Ar}), 136.78 (C_{Ar}), 135.95 (C_{Ar}), 131.12 (C_{Ar}), 129.36 (C-4/10), 129.06 (C_{Ar}), 128.93 (C-4/10), 20.99 (C-6/12), 20.94 (C-6/12), 19.32 (C-7/13), 19.23 (C-7/13), 0.30 (C-14);

3. Preparation of 2, 3, 4, 5, 6, 7

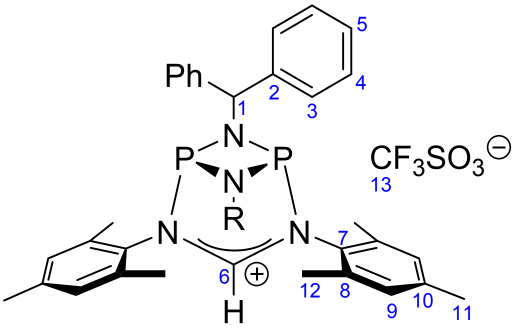

2

To a solution of **1** (2.00 g, 1.0 eq) in toluene was added a solution of the silylamidine (1.42 g, 1.0 eq) in toluene. The reaction mixture was left stirring for 30 min, then neat Et_3Si-OTf (913 µL, 1.0 eq) was added and the mixture was left stirring overnight. The supernatant was filtered off and the remaining colorless solid was washed with dry toluene and *n*-pentane. Drying *in vacuo* gave **2** (3.09 g, 90 %) as a colorless solid. Crystallization from dichloromethane by layering with toluene and *n*-pentane yielded colorless crystals which were washed with *n*-pentane and thoroughly dried under high vacuum. The single crystals obtained by this procedure are suitable for X-ray diffraction.

^{1}H-NMR (CD_2Cl_2, 600.13 MHz, 295 K): δ [ppm] = 7.85-7.82 (m, 1H, H-6), 7.69 (d, *J*= 7.7 Hz, 8H, H-3), 7.45 (t, *J*= 7.7 Hz, 8H, H-4), 7.36-7.33 (m, 4H, H-5), 6.92 (s, 4H, H-9), 5.59-5.56 (m, 2H, H-1), 2.25-2.22 (m, 18H, H-11 + H-12);

^{13}C{^{1}H}-NMR (CD_2Cl_2, 150.90 MHz, 295 K): δ [ppm] = 152.33 (s, C-6), 140.93 (s, C-10), 138.42 (m, C-2), 134.82 (m, C-7), 133.97 (s, C-8), 130.69 (s, C-9), 130.17 (s, C-4), 129.62 (s, C-5), 127.21 (s, C-3), 121.26 (q, *J*= 321.4 Hz, C-13), 64.60 (t, *J*= 6.8 Hz, C-1), 20.95 (s, C-11), 19.67 (s, C-12);

^{31}P{^{1}H}-NMR (CD_2Cl_2, 242.94 MHz, 295 K): δ [ppm] = 212.13 (s);

**Elemental analysis:**	found:	C 64.87%,	H 5.49%,	N 6.41%,
	calculated:	C 64.78%,	H 5.32%,	N 6.57%.

**MS (LIFDI(+)):**	m / z	703.2 ([M−OTf]$^+$)
	calculated:	703.3 ($C_{45}H_{45}N_4P_2$ $\hat{=}$ [M−OTf]$^+$)

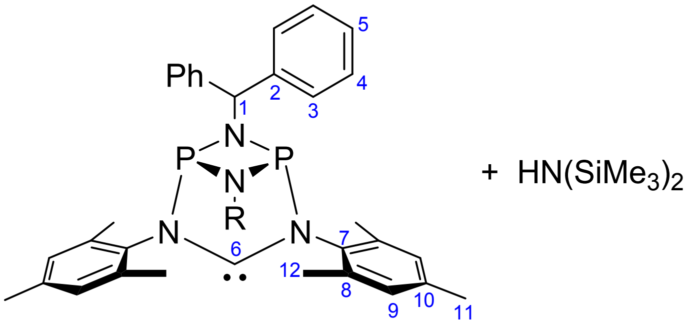

3

Toluene (3-5 mL) was added to a mixture of **2** (100 mg, 1.0 eq) and KHMDS (23.4 mg, 1.0 eq) at room temperature and the suspension was stirred for 10 min. The resulting yellow mixture was filtered and used in the next steps without further purification.

^{1}H-NMR (C_6D_6, 600.13 MHz, 295 K): δ [ppm] = 7.61-7.58 (m, 8H, H-3), 7.11-7.08 (m, 8H, H-4), 6.94-6.91 (m, 4H, H-5), 6.66 (s, 4H, H-9), 5.82 (t, *J*= 5.0 Hz, H-1), 2.44 (s, 12H, H-12), 2.02 (s, 6H, H-11);

^{13}C{^{1}H}-NMR (C_6D_6, 150.90 MHz, 295 K): δ [ppm] = 265.12 (m, C-6), 142.24 (m, C-7), 141.60 (m, C-2), 135.85 (s, C-10), 134.97 (s, C-8), 129.74 (s, C-9), 129.08 (s, C-4), 128.03 (s, C-5), 127.82 (s, C-3), 65.26 (t, *J*= 6.4 Hz, C-1), 20.80 (s, C-11), 20.42 (s, C-12);

^{31}P{^{1}H}-NMR (C_6D_6, 242.94 MHz, 295 K): δ [ppm] = 197.43 (s);

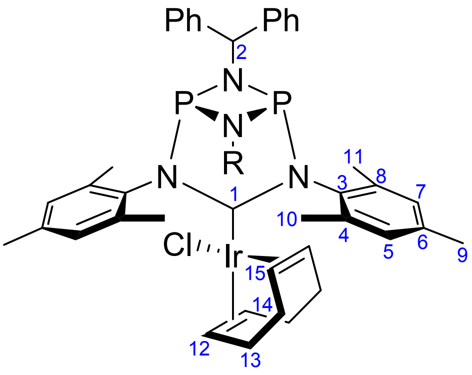

4

Toluene (3-5 mL) was added to a mixture of **2** (100 mg, 1.0 eq) and KHMDS (23.4 mg, 1 eq) at room temperature and the suspension was stirred for 10 min. Then, $[Ir(cod)Cl]_2$ (39.4 mg, 0.5 eq) was added and stirring was continued for 45 min. The resulting mixture was filtered, layered with *n*-pentane and stored at −40 °C. Thus, yellow/orange crystals were obtained, the supernatant was decanted, the crystals were washed with *n*-pentane and subsequently dried *in vacuo* to afford **4** (69.0 mg, 57 %). The crystals obtained by the above procedure are suitable for X-ray diffraction.

^{1}H-NMR (THF-d_8, 600.13 MHz, 295 K): δ [ppm] = 7.73-7.67 (m, 8H, H_{Ph}), 7.38-7.32 (m, 4H, H_{Ph}), 7.27-7.20 (m, 6H, H_{Ph}), 7.14-7.10 (m, 2H, H_{Ph}), 6.81 (s, 2H, H-5/7), 6.71 (s, 2H, H-5/7), 6.40-6.36 (m, 1H, H-2/2'), 5.69 (t, *J*= 5.0 Hz, 1H, H-2/2'), 4.08-4.03 (m, 2H, H-12/15), 2.89-2.84 (m, 2H, H-12/15), 2.33 (s, 6H, H-10/11), 2.20 (s, 6H, H-9), 2.05 (s, 6H, H-10/11), 1.37-1.17 (m, 4H, H-13/14), 1.16-1.08 (m, 4H, H-13/14);

^{13}C{^{1}H}-NMR (THF-d_8, 150.90 MHz, 295 K): δ [ppm] = 220.32 (m, C-1), 142.48 (m, *ipso*-C_{Ph}), 142.11 (m, *ipso*-C_{Ph}), 140.33 (m, C_{Mes}), 138.32 (s, C_{Mes}), 137.93 (s, C_{Mes}), 136.87 (s, C_{Mes}), 130.11 (s, C-5/7), 129.52 (s, C_{Ph}-H), 129.08 (s, C_{Ph}-H), 128.61 (s, C_{Ph}-H/C-5/7), 128.56 (s, C_{Ph}-H), 128.52 (s, C_{Ph}-H/C-5/7), 128.11 (s, C_{Ph}-H), 127.88 (s, C_{Ph}-H/C-5/7), 83.94 (s, C-12/15), 62.63 (m, C-2/2'), 53.72 (s, C-12/15), 33.97 (s, C-13/14), 28.22 (s, C-13/14), 22.10 (s, C-9/10/11), 21.57 (s, C-9/10/11), 20.61 (s, C-9/10/11), The ^{13}C resonance corresponding to the second benzhydryl carbon atom (C-2/2') is overlaid by the THF-d_8 resonance at 67.21 ppm.

^{31}P{^{1}H}-NMR (THF-d_8, 242.94 MHz, 295 K): δ [ppm] = 200.23 (s);

**Elemental analysis:**	found:	C 61.57%,	H 5.68%,	N 5.31%,
	calculated:	C 61.29%,	H 5.43%,	N 5.39%.

**MS (LIFDI(+)):**	m / z	1038.2	($[M]^+$)
	calculated:	1038.3	($C_{53}H_{56}N_4ClP_2Ir \hat{=} [M]^+$)

Cyclic Voltammetry: Conditions: inert gas atmosphere, solvent: dry CH_2Cl_2, 0.1 mol/L $n$$Bu_4NPF_6$, room temperature, scan rate: 100 mV s^{-1}, working electrode: glassy carbon, counter electrode: platinum, reference electrode: SCE, Fc/Fc^+ redox couple was used as an internal standard. (For the Fc/Fc^+ redox couple a value of 0.46 V vs. SCE was assumed.)[7]

Peak Potentials: +733 mV, +574 mV, ($E_{1/2}$=+0.653 V, ΔE=159 mV).

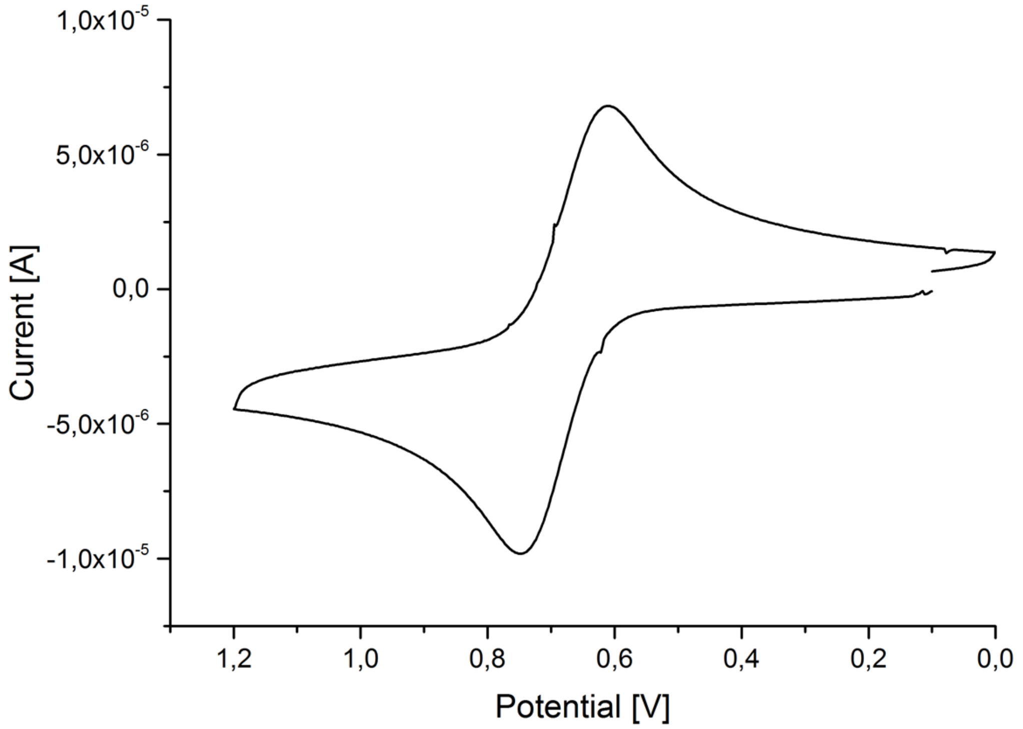

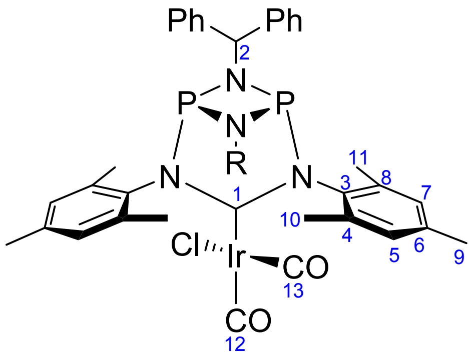

5

Reaction on NMR scale: $CO_{(g)}$ was bubbled through a solution of **4** in CH_2Cl_2 at room temperature for 5 min. The reaction is very fast and a color change (bright yellow) indicates the conversion. Evaporation of the solvent gave a quantitative yield of **5** as a bright yellow solid. Single crystals of **5** suitable for X-ray diffraction were obtained from a NMR solution of the compound in THF by careful layering with toluene and *n*-pentane and storage at −40 °C.

^{1}H-NMR (THF-d_8, 399.89 MHz, 298 K): δ [ppm] = 7.77-7.70 (m, 8H, H_{Ph}), 7.40-7.34 (m, 4H, H_{Ph}), 7.34-7.28 (m, 4H, H_{Ph}), 7.26-7.21 (m, 2H, H_{Ph}), 7.21-7.15 (m, 2H, H_{Ph}), 6.80 (m, 2H, H-5/7), 6.65 (m, 2H, H-5/7), 5.77-5.70 (m, 2H, H-2 + H-2'), 2.42 (s, 6H, H-9/10/11), 2.15 (s, 6H, H-9/10/11), 2.08 (s, 6H, H-9/10/11);

^{13}C{^{1}H}-NMR (THF-d_8, 100.55 MHz, 299 K): δ [ppm] = 207.68 (m, C-1), 181.21 (s, C-12/13), 171.14 (s, C-12/13), 141.53 (m, C_{Ph}), 141.25 (m, C_{Ph}), 139.56 (m, 2C, C_{Mes}), 138.55 (s, C_{Mes}), 136.99 (s, C_{Mes}), 135.74 (s, C_{Mes}), 130.09 (s, C-5/7), 129.72 (s, C_{Ph}H), 129.53 (m, 2C, C-5/7 + C_{Ph}H), 128.90 (s, C_{Ph}H), 128.52 (s, C_{Ph}H), 128.18 (m, C_{Ph}H), 127.89 (m, C_{Ph}H), 66.15 (t, *J*= 9.9 Hz, C-2/2'), 64.64 (t, *J*= 5.0 Hz, C-2/2'), 21.71 (m, C-9/10/11), 20.71 (s, C-9/10/11), 20.55 (s, C-9/10/11);

^{31}P{^{1}H}-NMR (THF-d_8, 242.94 MHz, 295 K): δ [ppm] = 202.00 (s);

**MS (LIFDI(+)):**	m / z	986.1	([M]$^+$)
	calculated:	986.2	($C_{47}H_{44}N_4P_2O_2IrCl$ ≙ [M]$^+$)

IR (CH_2Cl_2, room temperature): 1976 cm^{-1}, 2060 cm^{-1}.

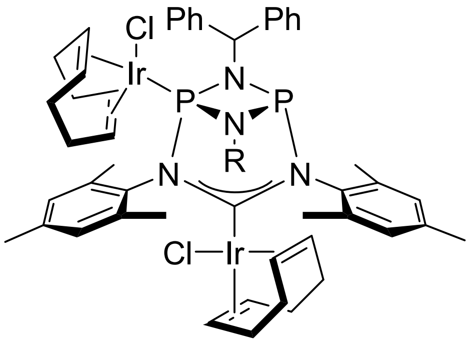

6

Toluene (3-5 mL) was added to a mixture of **2** (100 mg, 1.0 eq) and KHMDS (23.4 mg, 1.0 eq) at room temperature and the suspension was stirred for 10 min. Then, $[Ir(cod)Cl]_2$ (118.1 mg, 1.5 eq) was added and stirring was continued for 45 min. The resulting mixture was filtered, layered with *n*-pentane and stored at −40 °C. Thus, bright red crystals were obtained and the supernatant was decanted. The crystals were washed with *n*-pentane and thoroughly dried *in vacuo* to afford **6** (77.8 mg, 48 %). The crystals obtained by the above procedure are suitable for X-ray diffraction.

The NMR spectra shown below were taken at room temperature in the presence of a small excess of $[Ir(cod)Cl]_2$ to accumulate complex **6** in solution. Still, the spectra proved to be very complex due to significant overlay of signals.

^{1}H-NMR (THF-d_8, 600.13 MHz, 295 K): δ [ppm] = 8.05-8.02 (m, 2H, H_{Ar}), 7.64-7.60 (m, 2H, H_{Ar}), 7.58-7.55 (m, 2H, H_{Ar}), 7.61-7.46 (m, 1H, H_{Ar}), 7.40 (d, *J*= 7.6 Hz, 2H, H_{Ar}), 7.27-7.16 (m, 7H, Ph_2CH + H_{Ar}), 7.14-7.11 (m, 1H, H_{Ar}), 7.10-7.06 (m, 3H, H_{Ar}), 7.03-6.99 (m, 1H, H_{Ar}), 6.98-6.97 (m, 1H, H_{Mes}), 6.94-6.90 (m, 1H, Ph_2CH), 6.83-6.82 (m, 1H, H_{Mes}), 6.67-6.65 (m, 2H, H_{Mes}), 5.03-4.94 (m, 2H, H_{cod}), 4.25-4.15 (m, overlay with signals of excess $[Ir(cod)Cl]_2$), H_{cod}), 4.10-4.04 (m, 1H, H_{cod}), 3.15-3.09 (m, 1H, H_{cod}), 2.90-2.86 (m, 1H, H_{cod}), 2.82 (s, 3H, H_{Me}), 2.66-2.61 (m, 1H, H_{cod}), 2.39 (s, 3H, H_{Me}), 2.30 (s, 3H, H_{Me}), 2.18 (s, 3H, H_{Me}), 2.00-1.91 (m, 1H, H_{cod}), 1.83 (s, 3H, H_{Me}), 1.82-1.74 (m, 2H, H_{cod}), 1.71 (s, 3H, H_{Me}), 1.50-1.24 (m, 8H, H_{cod}), 1.22-1.16 (m, 1H, H_{cod}), 1.15-0.99 (m, 4H, H_{cod}), 0.98-0.91 (m, 1H, H_{cod});

^{13}C{^{1}H}-NMR (CD_2Cl_2, 150.90 MHz, 295 K): δ [ppm] = 228.04 (m, C-1), 144.58 (d, *J*= 5.8 Hz, C_{Ar}), 143.74 (d, *J*= 2.2 Hz, C_{Ar}), 142.30 (m, C_{Ar}), 142.12 (d, *J*= 12.3 Hz, C_{Ar}), 141.61 (m, C_{Ar}), 41.12 (d, *J*= 7.8 Hz, C_{Ar}), 140.27 (d, *J*= 15.5 Hz, C_{Ar}), 138.64 (d, *J*= 2.5 Hz, C_{Ar}), 138.15 (d, *J*= 1.9 Hz, C_{Ar}), 138.09 (m, C_{Ar}), 137.99 (d, *J*= 3.0 Hz, C_{Ar}), 137.91 (d, *J*= 1.6 Hz, C_{Ar}), 130.31-130.04 (m, multiple carbon atoms, C_{Ar}-H), 129.76-129.67 (m, multiple carbon atoms, C_{Ar}-H), 129.70 (s, C_{Ar}-H), 129.37 (s, C_{Ar}-H), 129.32-129.26 (m, multiple carbon atoms, C_{Ar}-H), 129.03 (s, C_{Ar}-H), 128.87-128.67 (m, multiple carbon atoms, C_{Ar}-H), 128.38 (s, C_{Ar}-H), 128.19

(s, C_{Ar}-H), 128.07 (s, C_{Ar}-H), 127.44 (s, C_{Ar}-H), 108.42 (d, *J*= 14.4 Hz, C_{cod}), 104.60 (d, *J*= 18.3 Hz, C_{cod}), 84.88 (s, C_{cod}), 83.40 (s, C_{cod}), 64.25 (t, *J*= 6.0 Hz, Ph_2*C*H), 63.31 (m, Ph_2*C*H), 60.07 (s, C_{cod}), 57.15 (s, C_{cod}), 54.36 (s, C_{cod}), 48.43 (s, C_{cod}), 36.76 (d, *J*= 3.6 Hz, CH_2), 34.06 (s, CH_2), 33.69 (s, CH_2), 31.48 (s, CH_2), 30.88 (s, CH_2), 28.23 (s, CH_2), 28.21 (s, CH_2), 25.46 (s, CH_3), 25.45 (s, CH_3), 25.33 (s, CH_2), 24.79 (s, CH_3), 22.40 (s, CH_3), 20.82 (s, CH_3), 20.48 (s, CH_3);

^{31}P-NMR (THF-d_8, 242.94 MHz, 295 K): δ [ppm] = 162.28 (broadened signal, 1P), 158.42 (d, *J*= 30.5 Hz, 1P);

**Elemental analysis:**	found:	C 53.38%,	H 5.02%,	N 4.13%,
	calculated:	C 53.30%,	H 4.99%,	N 4.08%.

**MS (LIFDI(+)):**	m / z	1339.5 ($[M-Cl]^+$)
	calculated:	1339.4 ($C_{61}H_{68}N_4P_2Ir_2Cl_2$ $\hat{=}$ $[M-Cl]^+$)

The $^{31}P\{^1H\}$ spectrum below shows compound **6** in solution (THF) at room temperature in the presence of an excess of $[Ir(cod)Cl]_2$. A small amount of monometallic complex **4** can still be detected at 200.23 ppm.

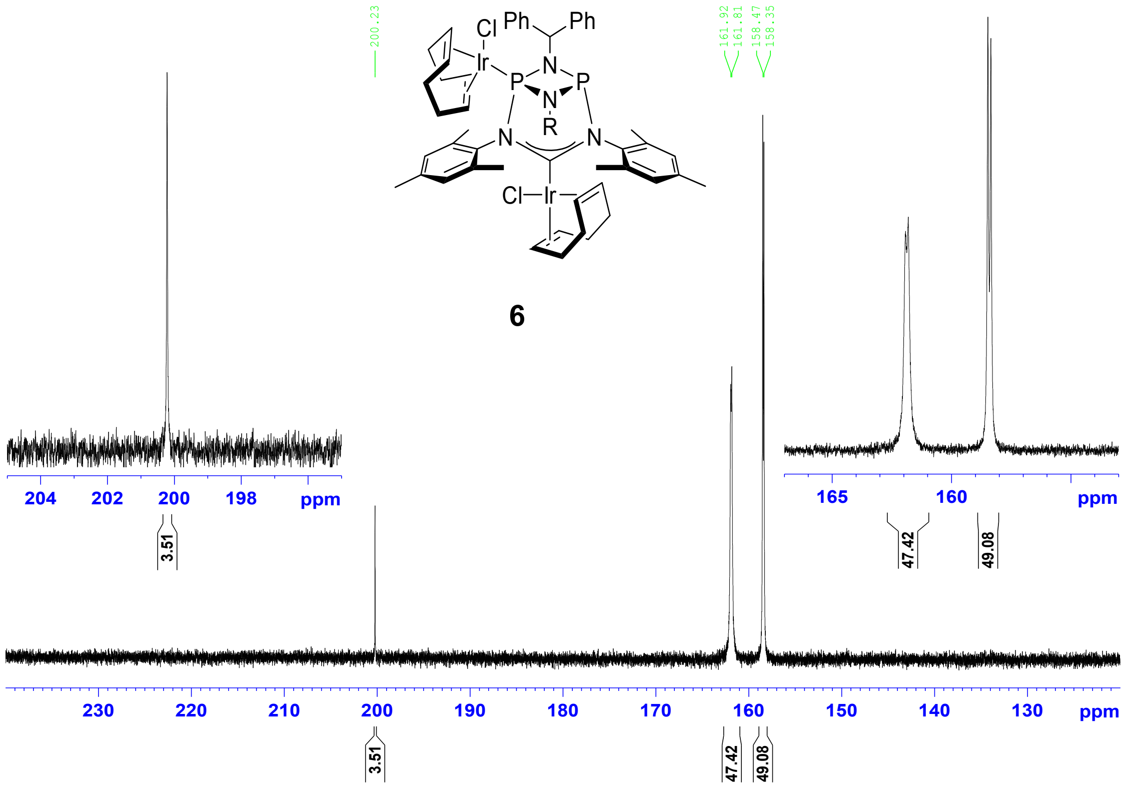

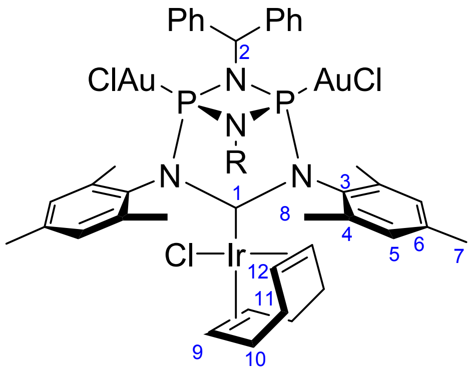

7

Toluene (3-5 mL) was added to a mixture of **2** (100.0 mg, 1.0 eq) and KHMDS (23.4 mg, 1.0 eq) at room temperature and the suspension was stirred for 10 min. Then, $[Ir(cod)Cl]_2$ (39.4 mg, 0.5 eq) was added and stirring was continued for 1 $(Me_2S)AuCl$ (103.6 mg, 3.0 eq) was added and the mixture was left stirring for 1 h. After filtration the clear red solution was carefully layered with *n*-pentane and stored at −40 °C. After precipitation a bright red/brown powder was obtained and the supernatant was decanted. The solid was washed with *n*-pentane and thoroughly dried *in vacuo* to afford **7** (81.2 mg, 46 %). Orange single crystals suitable for X-ray diffraction were obtained by layering a solution of the compound in toluene with *n*-pentane.

^{1}H-NMR (CD_2Cl_2, 399.89 MHz, 297 K): δ [ppm] = 7.81-7.76 (m, 8H, H_{Ph}), 7.53-7.47 (m, 4H, H_{Ph}), 7.46-7.30 (m, 8H, H_{Ph}), 6.92 (s, 2H, H-5), 6.88 (s, 2H, H-5'), 6.82-6.80 (m, 1H, H-2), 5.90 (t, *J*= 7.7 Hz, 1H, H-2'), 4.47-4.40 (m, 2H, H-9/12), 2.81-2.74 (m, 2H, H-9/12), 2.29 (s, 6H, H-7/7'/8), 2.28 (s, 6H, H-7/7'/8), 2.20 (s, 6H, H-7/7'/8), 1.45-1.16 (m, 8H, H-10 + H-11);

^{13}C{^{1}H}-NMR (CD_2Cl_2, 150.90 MHz, 295 K): δ [ppm] = 233.78 (t, *J*= 4.5 Hz, C-1), 140.54 (s, C_{Ar}), 138.52 (t, *J*= 5.7 Hz, C_{Ar}), 138.40 (t, *J*= 4.0 Hz, C_{Ar}), 137.84 (m, C_{Ar}), 137.37 (t, *J*= 4.9 Hz, C_{Ar}), 135.90 (m, C_{Ar}), 130.71 (s, C_{Ar}), 130.48 (s, C_{Ph}), 130.10 (s, C_{Ph}), 130.05 (s, C_{Ar}), 129.19 (s, C_{Ar}), 129.09 (s, C_{Ar}), 128.91 (s, C_{Ph}), 128.11 (s, C_{Ph}), 91.04 (s, C-9/12), 68.72 (m, C-2), 63.59 (s, C-2), 58.20 (s, C-9/12), 33.36 (s, C-10/11), 27.70 (s, C-10/11), 23.00 (s, C-7/8/8'), 21.69 (s, C-7/8/8'), 20.98 (s, C-7/8/8');

^{31}P{^{1}H}-NMR (CD_2Cl_2, 161.88 MHz, 298 K): δ [ppm] = 127.65 (s);

**Elemental analysis:**	found:	C 42.47%,	H 4.01%,	N 3.70%,
	calculated:	C 42.34%,	H 3.75%,	N 3.73%.

**MS (LIFDI(+)):**	m / z	1501.8	$([M]^+)$
	calculated:	1502.2	$(C_{53}H_{56}N_4Cl_3P_2IrAu_2 \hat{=} [M]^+)$

4. Variable Temperature Experiments

For this experiment single crystals of **6** were dissolved in THF-d_8.

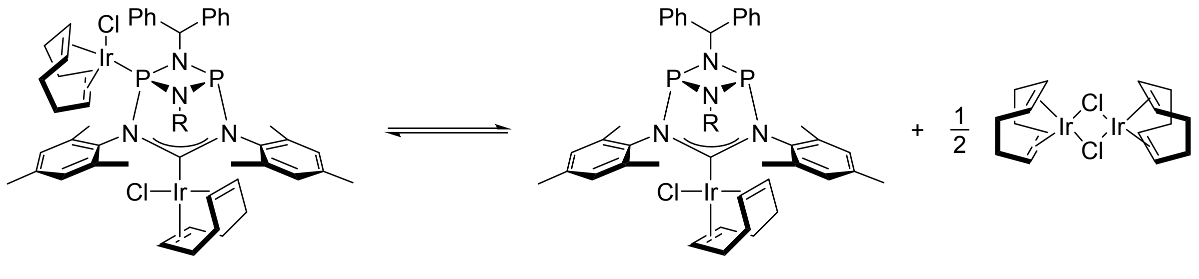

Table S1. Determination of K for different temperatures T.

T [K]	K	1/T [K^{-1}]	ln(K)
243	0,0038964	0,0041	-5,5477
258	0,0069437	0,0039	-4,9699
273	0,0146475	0,0037	-4,2235
283	0,0213712	0,0035	-3,8457
295	0,0444624	0,0034	-3,1131
308	0,0755426	0,0032	-2,5831
323	0,1519063	0,0031	-1,8845

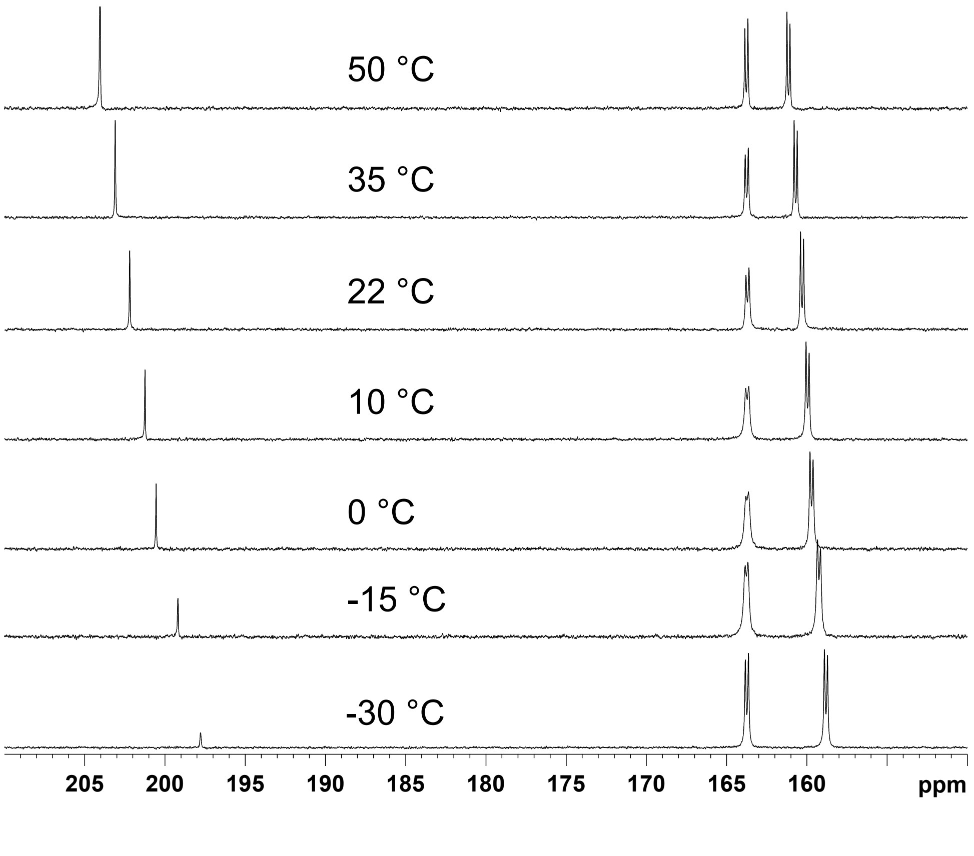

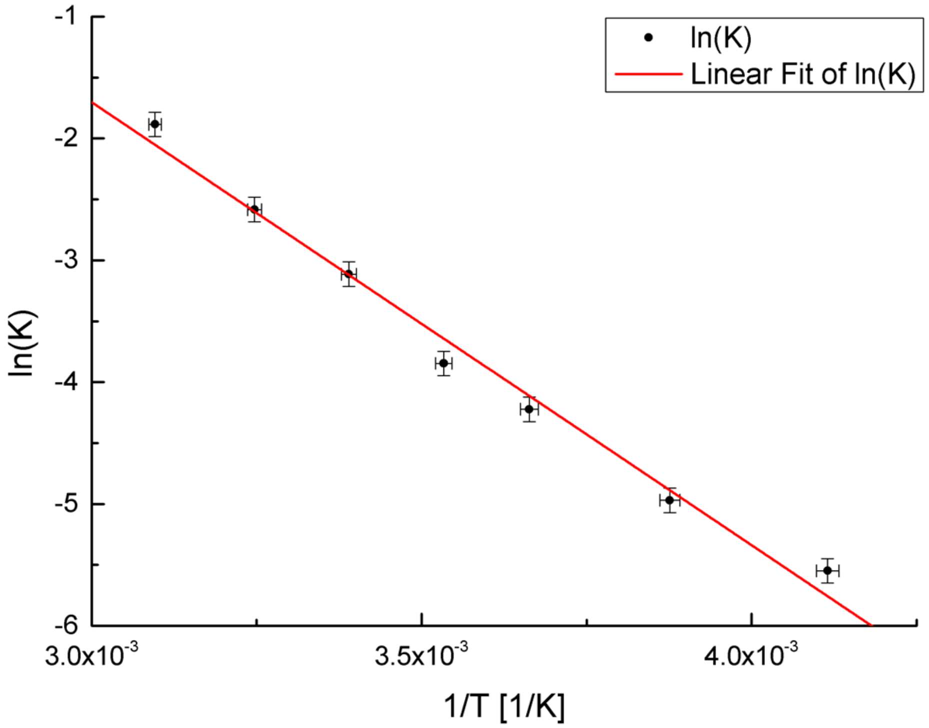

Estimated error in the temperature T: 1 K; estimated error of the equilibrium constant K: 1 %

Intercept: 9.2034 ± 0.6666

Slope: −3635.2269 ± 186.4568 K

$y = m \cdot x + b = -3635.2269 \cdot x + 9.2034$

$\Delta H = -m \cdot R = 30 \pm 6 \text{ kJ mol}^{-1}$

$\Delta S = b \cdot R = 77 \pm 2 \text{ J mol}^{-1}$

5. X-ray Crystal Structure Determinations

Crystal data and details of the structure determinations are listed in Table S2. Full shells of intensity data were collected at low temperature with a Agilent Technologies Supernova-E CCD diffractometer (Mo- or Cu-K_a radiation, microfocus tube, multilayer mirror optics). Data were corrected for air and detector absorption, Lorentz and polarization effects;[8] absorption by the crystal was treated numerically (Gaussian grid)[8] or with a semiempirical multiscan method (all other complexes).[9] The structures were solved by the charge flip procedure[10] and refined by full-matrix least squares methods based on F^2 against all unique reflections.[11] Hydrogen atoms were generally input at calculated positions and refined with a riding model. When justified by the quality of the data the positions of some hydrogen atoms (in complex **4** those on the carbon atoms involved in coordination to Ir) were taken from difference Fourier syntheses and refined. When found necessary, disordered groups and/or solvent molecules where subjected to suitable geometry and adp restraints or constraints. Due to severe disorder and/or fractional occupancy, electron density attributed to solvent of crystallization was removed from the structures of **5**, **6** and **7** with the BYPASS procedure,[12] as implemented in PLATON (SQUEEZE).[13] Partial structure factors from the solvent masks were included in the refinement as separate contributions to F_{obs}.

In the structure of **5** there is evidence for an unresolvable minor positional disorder (approx. 10 %) of the chloride on Ir and the carbonyl ligand *trans* to Cl. As a consequence, the adp elipsoids of C(47) and O(2) appear elongated along the Ir-C-O vector, and C(47) is pushed towards O(2) during refinement, leading to an unrealistically short distance C(47)-O(2). In order to battle this effect and the additional C-O "sliding" caused by anisotropic refinement[14] the distances Ir-C(47) and C(47)-O(2) were restrained to sensible values (1.856 Å and 1.13 Å, respectively, taken from an analysis of 12 comparable low temperature structures in the CCDC database).[15]

Table S2. Details of the crystal structure determinations.

	2	4	5	6	7
formula	$C_{53}H_{53}F_3N_4O_3P_2S$	$C_{53}H_{56}IrN_4P_2{\cdot}2C_7H_8$	$C_{47}H_{44}ClIrN_4O_2P_2{\cdot}C_7H_8$	$C_{61}H_{68}Cl_2Ir_2N_4P_2{\cdot}1.5C_7H_8$	$C_{53}H_{56}Au_2Cl_3IrN_4P_2{\cdot}0.5C_5H_{12}$
M_r	944.99	1222.87	1078.58	1512.13	1539.51
crystal system	monoclinic	triclinic	trigonal	triclinic	monoclinic
space group	Cc (IT Nr. 9)	P -1 (IT Nr. 2)	R -3 (IT Nr. 148)	P -1 (IT Nr. 2)	P 21/c (IT Nr. 14)
a /Å	24.05898(14)	11.53121(12)	30.1754(4)	13.28816(19)	12.88426(10)
b /Å	12.60445(6)	15.09347(18)		15.4534(2)	13.46559(9)
c /Å	17.26646(9)	16.48026(17)	27.5094(4)	18.6095(2)	30.9781(2)
α /°		91.4726(9)		66.4438(13)	
β /°	109.8994(6)	100.9553(9)		87.7181(11)	99.5872(7)
γ /°		90.9436(9)		64.8361(15)	
V /Å^3	4923.42(5)	2814.45(5)	21693.0(6)	3131.27(9)	5299.46(7)
Z	4	2	18	2	4
F_{000}	1984	1252	9792	1509	2956
d_c /Mg·m^{-3}	1.275	1.443	1.486	1.604	1.930
X-radiation, λ /Å	Cu K_α 1.54184	Mo K_α 0.71073	Mo K_α 0.71073	Mo K_α 0.71073	Mo K_α 0.71073
μ /mm^{-1}	1.676	2.522	2.937	4.428	8.281
max., min. transmission factors	0.912, 0.819	0.938, 0.673	0.862, 0.758	0.762, 0.535	0.780, 0.268
data collect. temperat. /K	120(1)	120(1)	120(1)	120(1)	120(1)
θ range /°	3.9 to 70.8	3.3 to 32.9	3.2 to 29.0	3.2 to 32.9	3.2 to 32.2
index ranges *h*,*k*,*l*	-29 ... 28, -15 ... 15, -21 ... 21	-17 ... 17, -22 ... 22, -25 ... 24	-40 ... 40, -39 ... 41, -37 ... 37	-20 ... 19, -23 ... 23, -28 ... 28	-19 ... 19, -20 ... 20, -45 ... 46
reflections measured	119832	210789	256938	205422	315804
unique [R_{int}]	9108 [0.0415]	19792 [0.0742]	12550 [0.0832]	21933 [0.0739]	18285 [0.0526]
observed [$I \geq 2\sigma(I)$]	8965	18429	10220	18820	16689
parameters refined	627	696	520	698	592
GooF on F^2	1.034	1.118	1.035	1.111	1.333
R indices [$F>4\sigma(F)$] $R(F)$, $wR(F^2)$	0.0418, 0.1113	0.0357, 0.0730	0.0347, 0.0752	0.0408, 0.0761	0.0356, 0.0829
R indices (all data) $R(F)$, $wR(F^2)$	0.0425, 0.1120	0.0410, 0.0747	0.0497, 0.0794	0.0527, 0.0798	0.0414, 0.0843
absolute structure parameter	0.011(4)				
Difference density: max, min /e·Å^{-3}	0.046, 0.603, -0.419	0.121, 2.340, -1.738	0.104, 1.070, -0.927	0.145, 2.121, -1.727	0.201, 2.636, -1.643

7. Solid State Structures

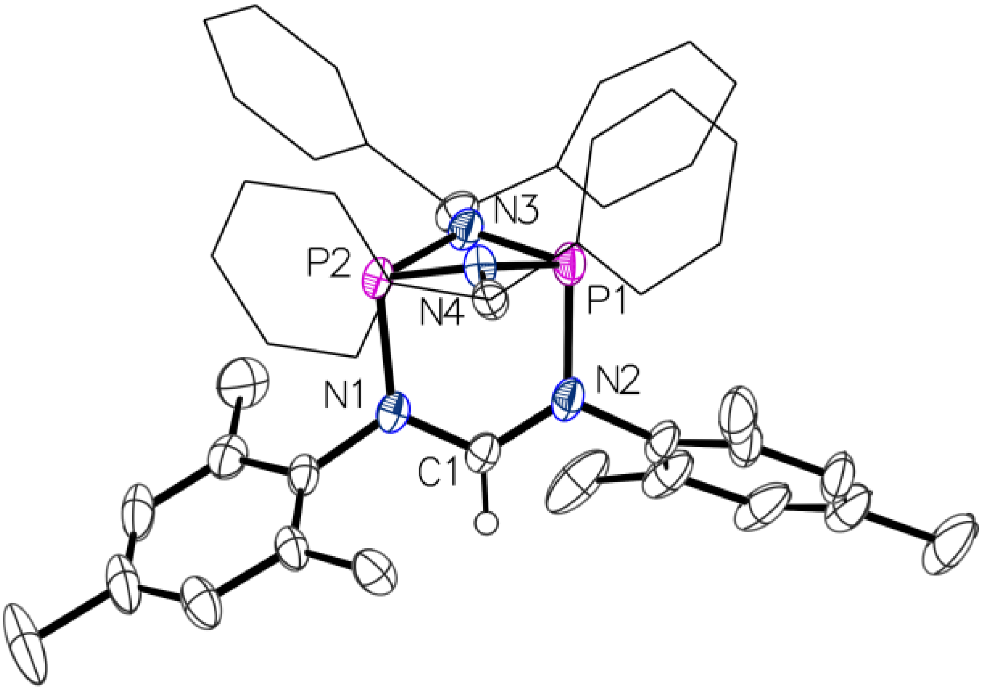

Molecular structure of compound **2**, thermal ellipsoids set at the 50 % probability level. H-atoms except for H(1), a cocrystallized solvent molecule (toluene) and a second orientation of the disordered trifluoromethanesulfonate group are omitted for clarity. Phenyl rings of the benzhydryl substituents are drawn as wireframes.Selected bond lengths [Å] and angles [°]: P(1)-N(2) 1.837(3), P(1)-N(3) 1.704(3), P(1)-N(4) 1.709(3), P(2)-N(1) 1.823(3), P(2)-N(3) 1.716(3), P(2)-N(4) 1.708(3), N(1)-C(1) 1.323(4), N(2)-C(1) 1.322(5), N(1)-P(2)-P(1) 86.91(9), N(4)-P(2)-N(3) 80.34(14), N(3)-P(2)-N(1) 98.88(13), C(1)-N(1)-P(2) 122.0(2), C(1)-N(2)-P(1) 121.9(2), P(1)-N(3)-P(2) 95.63(14), N(2)-C(1)-N(1) 122.7(3), selected torsion angles [°]: P(1)-P(2)-N(1)-C(1) -2.3(2), N(3)-P(1)-N(4)-P(2) 20.84(14).

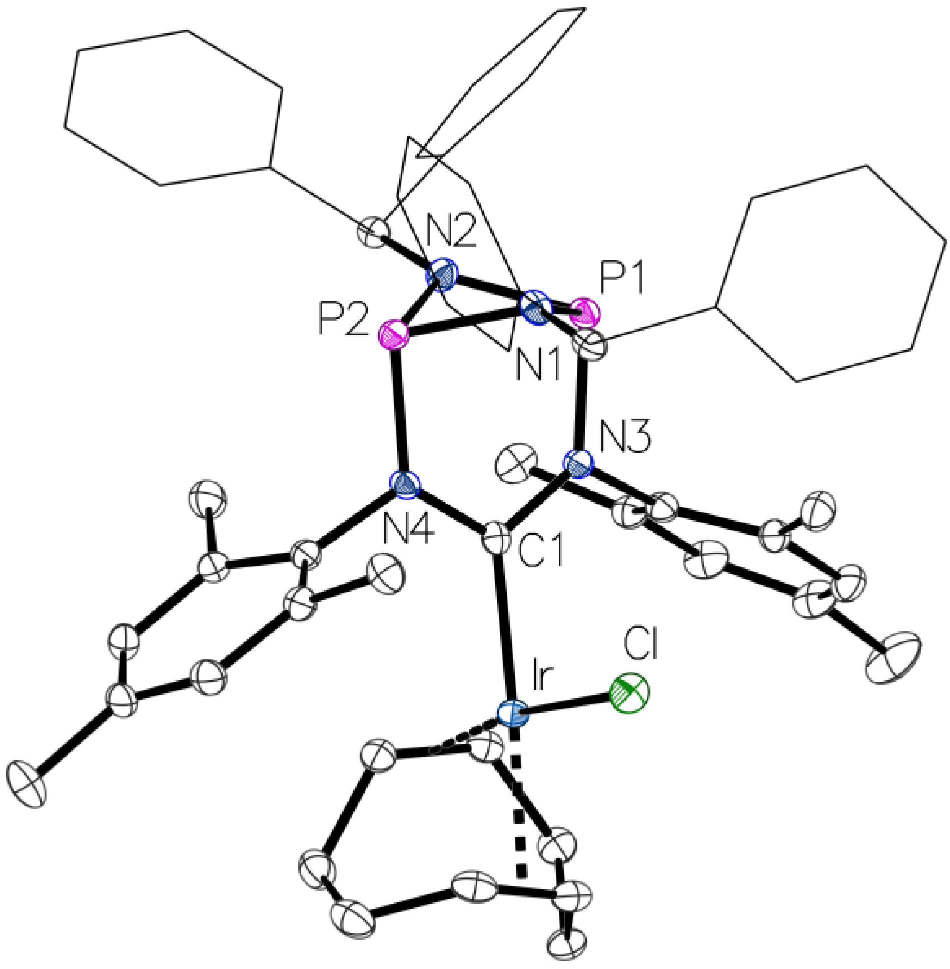

Molecular structure of compound **4**, thermal ellipsoids set at the 50 % probability level. H-atoms and two solvent molecules (toluene) are omitted for clarity. Phenyl rings of the benzhydryl substituents are drawn as wireframes. Selected bond lengths [Å] and angles [°]: Ir-Cl 2.3812(6), Ir-C(1) 2.040(2), P(1)-N(1) 1.727(2), P(1)-N(2) 1.726(2), P(1)-N(3) 1.7658(19), P(2)-N(1) 1.731(2), P(2)-N(2) 1.720(2), P(2)-N(4) 1.7752(19), N(3)-C(1) 1.372(3), N(4)-C(1) 1.381(3), C(1)-Ir-Cl 87.99(6), N(1)-P(1)-N(3) 99.79(9), N(2)-P(1)-N(1) 79.82(10), N(2)-P(1)-N(3) 101.05(9), P(1)-N(1)-P(2) 94.08(10), C(1)-N(3)-P(1) 126.57(15), N(3)-C(1)-Ir 124.06(16), N(3)-C(1)-N(4) 114.06(19), N(4)-C(1)-Ir 121.30(15), selected torsion angles [°]: P(1)-N(3)-C(1)-Ir -165.18(11), P(1)-N(3)-C(1)-N(4) 6.2(3), N(1)-P(1)-N(2)-P(2) -25.66(9), N(1)-P(1)-N(3)-C(1) 37.3(2), N(4)-P(2)-N(1)-P(1) 74.27(10);

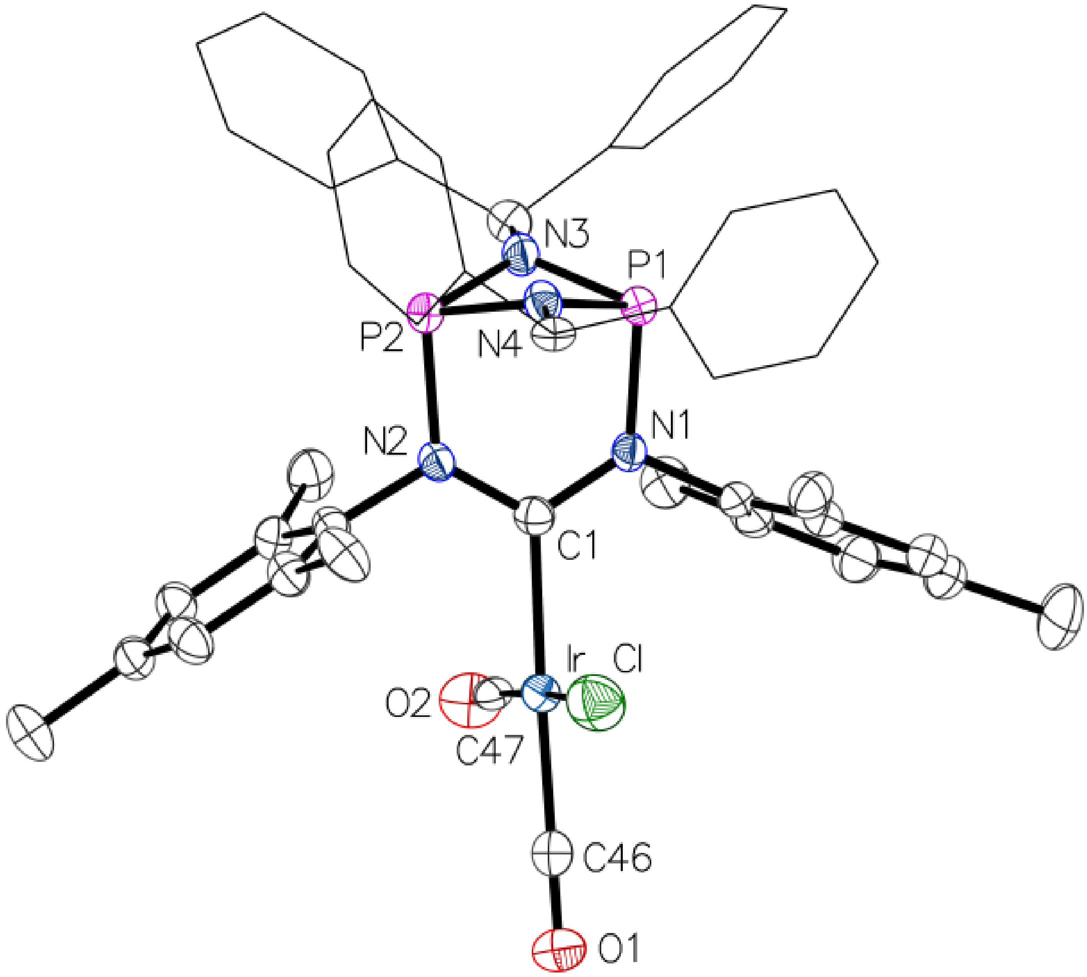

Molecular structure of compound **5**, thermal ellipsoids set at the 50 % probability level. H-atoms are omitted for clarity. Phenyl rings of the benzhydryl substituents are drawn as wireframes. Selected bond lengths [Å] angles [°]: Ir-Cl 2.3414(10), Ir-C(1) 2.107(3), Ir-C(46) 1.891(3), Ir-C(47) 1.855,[a] P(1)-N(1) 1.790(2), P(1)-N(3) 1.725(3), O(1)-C(46) 1.141(4), O(2)-C(47) 1.123,[a] N(1)-C(1) 1.352(4), N(2)-C(1) 1.357(4), C(1)-Ir-Cl 86.49(9), C(46)-Ir-C(1) 173.88(14), N(1)-P(1)-P(2) 86.30(8), N(4)-P(1)-N(3) 79.82(13), C(1)-N(1)-P(1) 125.6(2), P(1)-N(4)-P(2) 94.57(13), N(1)-C(1)-Ir 121.2(2), N(1)-C(1)-N(2) 116.4(3), selected torsion angles [°]:P(1)-N(1)-C(1)-Ir -168.80(14), P(1)-N(1)-C(1)-N(2) 5.0(4), N(3)-P(1)-N(4)-P(2) 24.79(13), N(3)-P(1)-N(1)-C(1) -45.9(3).

[a] distance restrained.

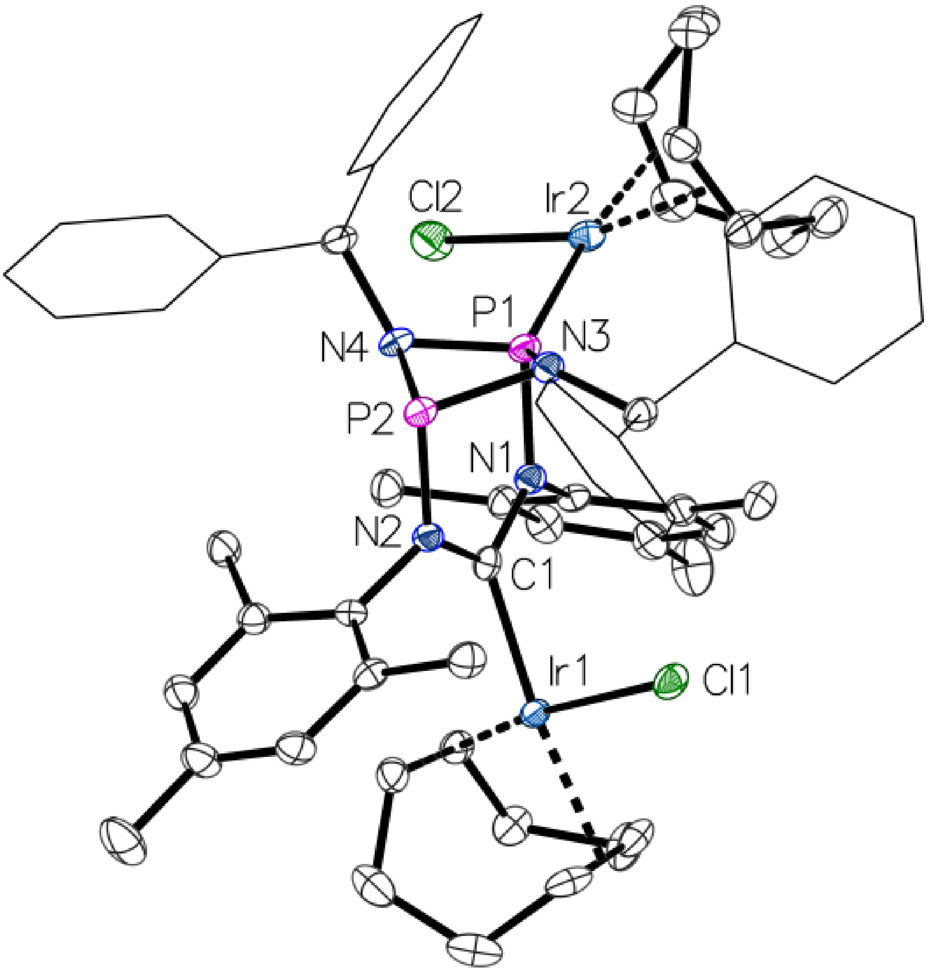

Molecular structure of compound **6**, thermal ellipsoids set at the 50 % probability level. H-atoms and a cocrystallized solvent molecule (toluene) are omitted for clarity. Phenyl rings of the benzhydryl substituents are drawn as wireframes. Selected bond lengths [Å] and angles [°]: Ir(1)-Cl(1) 2.3768(8), Ir(1)-C(1) 2.046(3), Ir(2)-Cl(2) 2.3659(9), Ir(2)-P(1) 2.2359(8), P(1)-N(1) 1.756(3), P(1)-N(3) 1.743(3), P(1)-N(4) 1.707(3), P(2)-N(2) 1.760(3), P(2)-N(3) 1.744(3), P(2)-N(4) 1.745(3), N(1)-C(1) 1.385(4), N(2)-C(1) 1.376(4), C(1)-Ir(1)-Cl(1) 86.21(8), P(1)-Ir(2)-Cl(2) 93.14(3), Ir(2)-P(1)-P(2) 158.50(4), N(1)-P(1)-Ir(2) 111.96(9), N(1)-P(1)-P(2) 87.88(9), N(4)-P(1)-N(3) 84.19(13), N(3)-P(2)-N(4) 83.04(13), C(1)-N(1)-P(1) 124.4(2), C(1)-N(2)-P(2) 125.6(2), P(1)-N(3)-P(2) 91.35(13), P(1)-N(4)-P(2) 92.49(13), N(1)-C(1)-Ir(1) 121.1(2), N(2)-C(1)-Ir(1) 122.7(2), N(2)-C(1)-N(1) 114.9(3), selected torsion angles [°]: Ir(2)-P(1)-N(1)-C(1) -172.9(2), Ir(2)-P(1)-N(3)-P(2) 149.69(7), Ir(2)-P(1)-N(4)-P(2) -160.65(6), P(1)-P(2)-N(2)-C(1) 7.6(2), P(1)-N(1)-C(1)-Ir(1) -160.75(15), P(1)-N(1)-C(1)-N(2) 6.6(4), P(2)-P(1)-N(1)-C(1) -1.5(2), P(2)-N(2)-C(1)-Ir(1) 156.84(16), P(2)-N(2)-C(1)-N(1) -10.3(4), N(1)-P(1)-N(3)-P(2) -76.77(13), N(3)-P(1)-N(1)-C(1) 41.4(3), N(3)-P(1)-N(4)-P(2) -22.46(12), N(3)-P(2)-N(4)-P(1) 22.49(12), N(4)-P(1)-N(3)-P(2) 22.46(12).

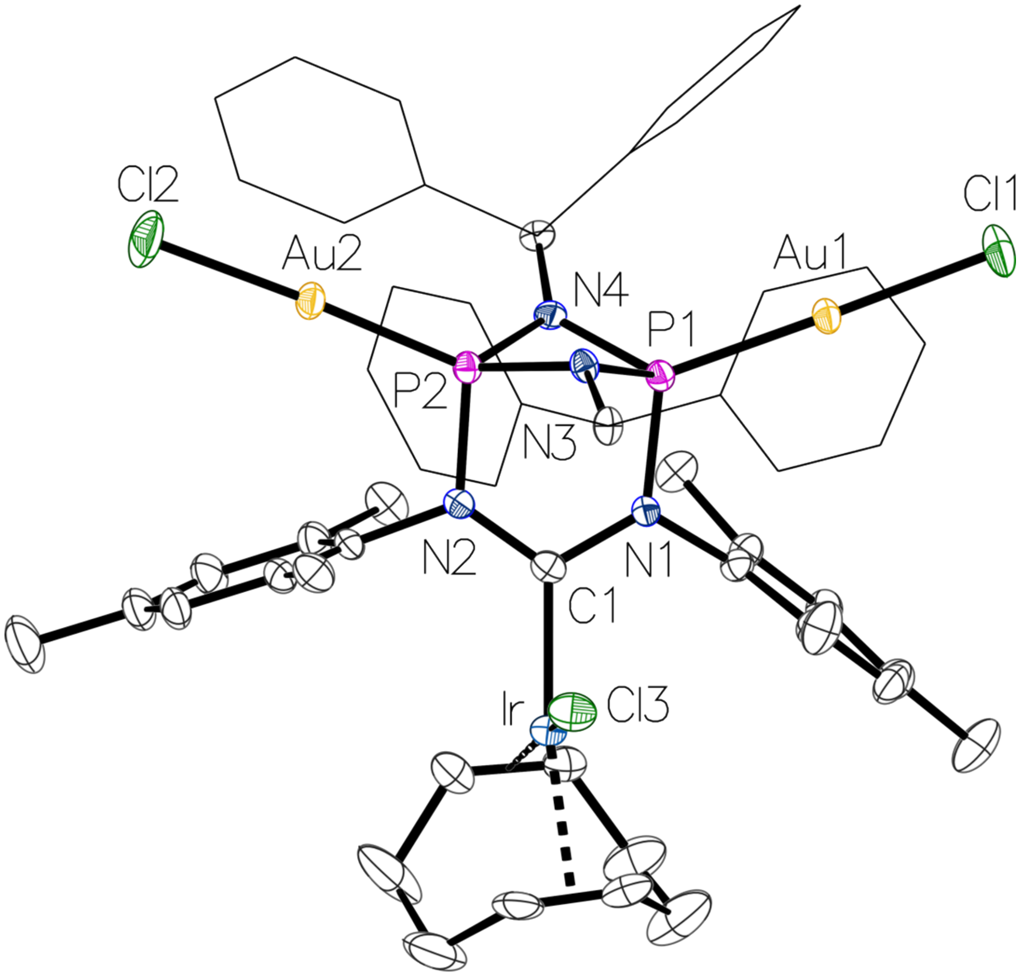

Molecular structure of compound **7**, thermal ellipsoids set at the 50 % probability level. H-atoms are omitted for clarity. Phenyl rings of the benzhydryl substituents are drawn as wireframes. Selected bond lengths [Å] and angles [°]: Au(1)-Cl(1) 2.2730(11), Au(1)-P(1) 2.1839(10), Au(2)-Cl(2) 2.2739(12), Au(2)-P(2) 2.1862(10), Ir-Cl(3) 2.3741(12), Ir-C(1) 2.020(4), P(1)-N(1) 1.719(4), P(1)-N(3) 1.712(4), N(1)-C(1) 1.390(5), P(1)-Au(1)-Cl(1) 177.39(4), P(2)-Au(2)-Cl(2) 177.04(5), C(1)-Ir-Cl(3) 86.80(12), Au(1)-P(1)-P(2) 156.77(6), N(1)-P(1)-P(2) 89.16(12), N(3)-P(1)-N(1) 101.38(17), Au(2)-P(2)-P(1) 159.68(6), N(4)-P(1)-N(3) 82.48(17), C(1)-N(1)-P(1) 123.2(3), P(1)-N(3)-P(2) 90.14(17), N(2)-C(1)-N(1) 115.3(4), selected torsion angles [°]: P(1)-N(1)-C(1)-Ir -161.3(2), P(2)-P(1)-N(1)-C(1) -3.1(3), P(2)-N(2)-C(1)-N(1) -8.2(5), N(3)-P(1)-N(1)-C(1) 40.5(4), N(3)-P(1)-N(4)-P(2) -27.16(17);

8. Space-filling representations of the donor sites

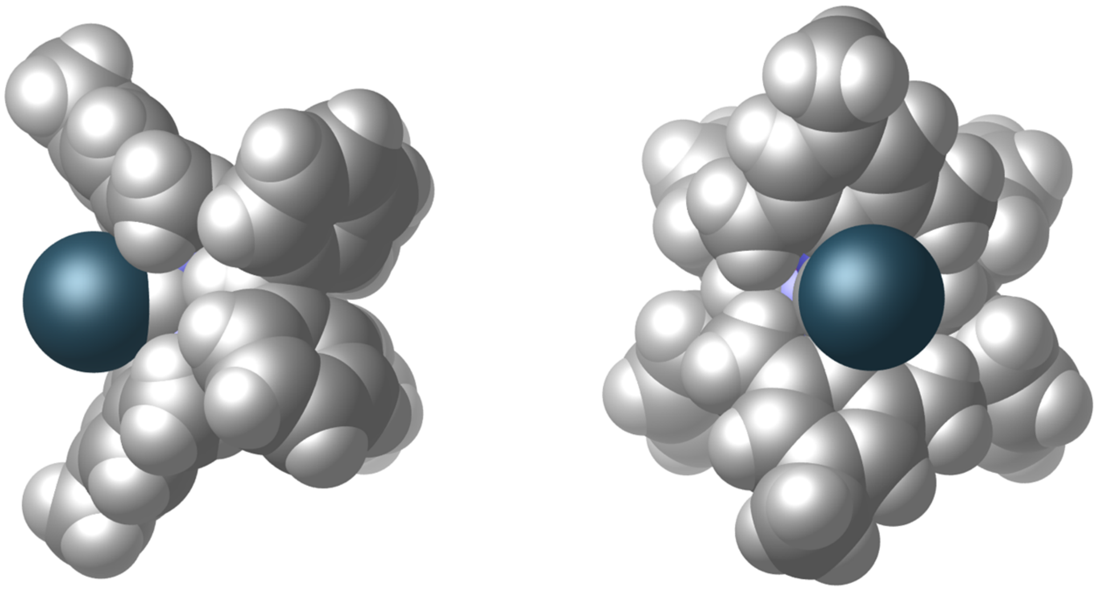

Side view (left) and front view (right) of the NHC-Ir moiety shown as a space-filling representation.

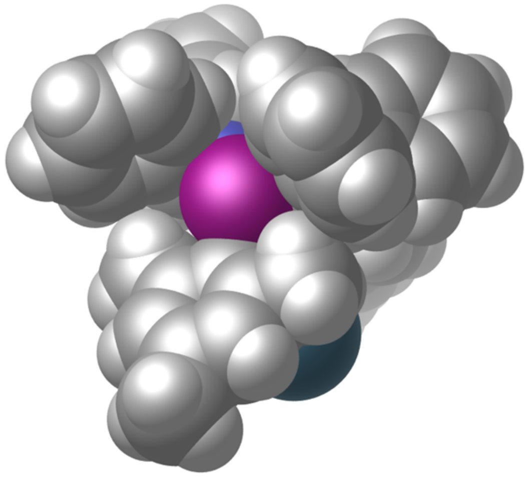

Space-filling representation of one of the C_3-symmetric phosphorus-centred binding pockets. The cavity is enclosed by two benzhydryl phenyl and a mesityl ring.

9. Selected NMR Spectra

^{1}H, $^{31}P\{^{1}H\}$ and $^{13}C\{^{1}H\}$ NMR spectra of single crystals of cationic cage **2** in dichloromethane-d_2.

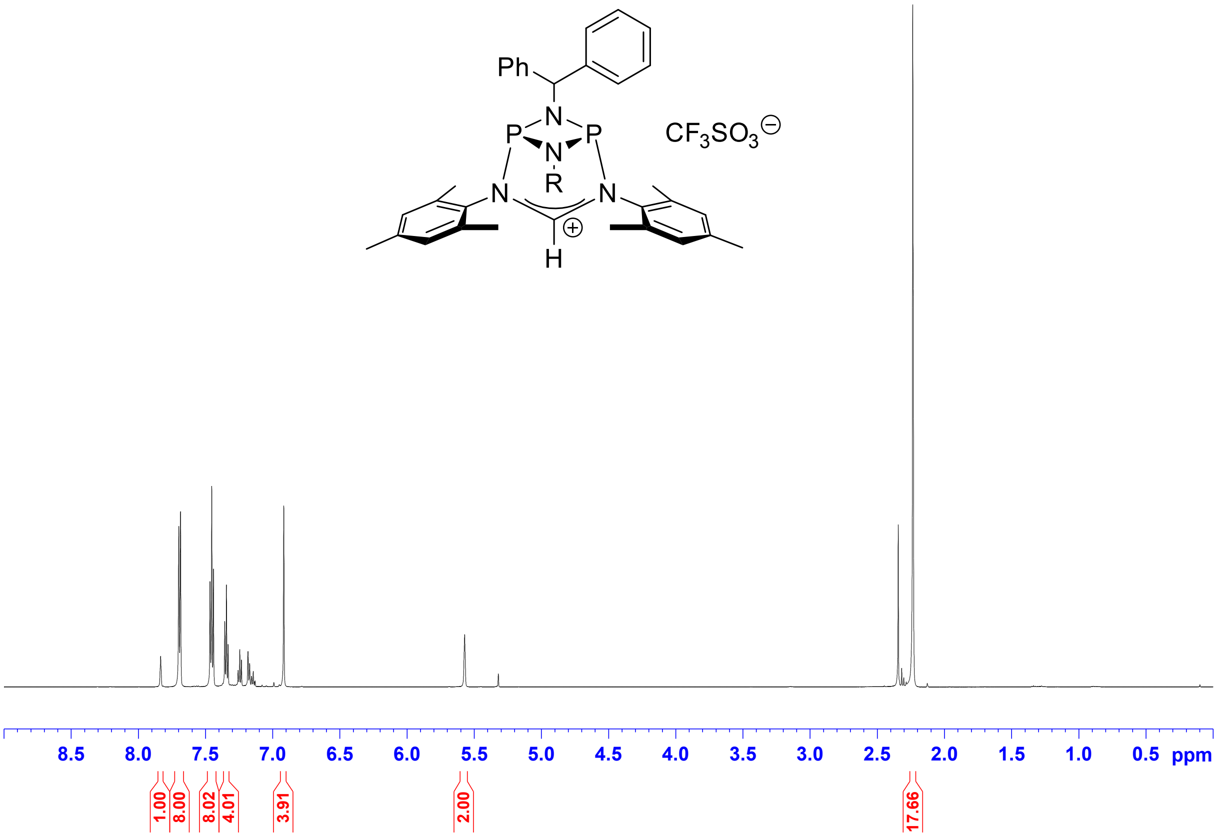

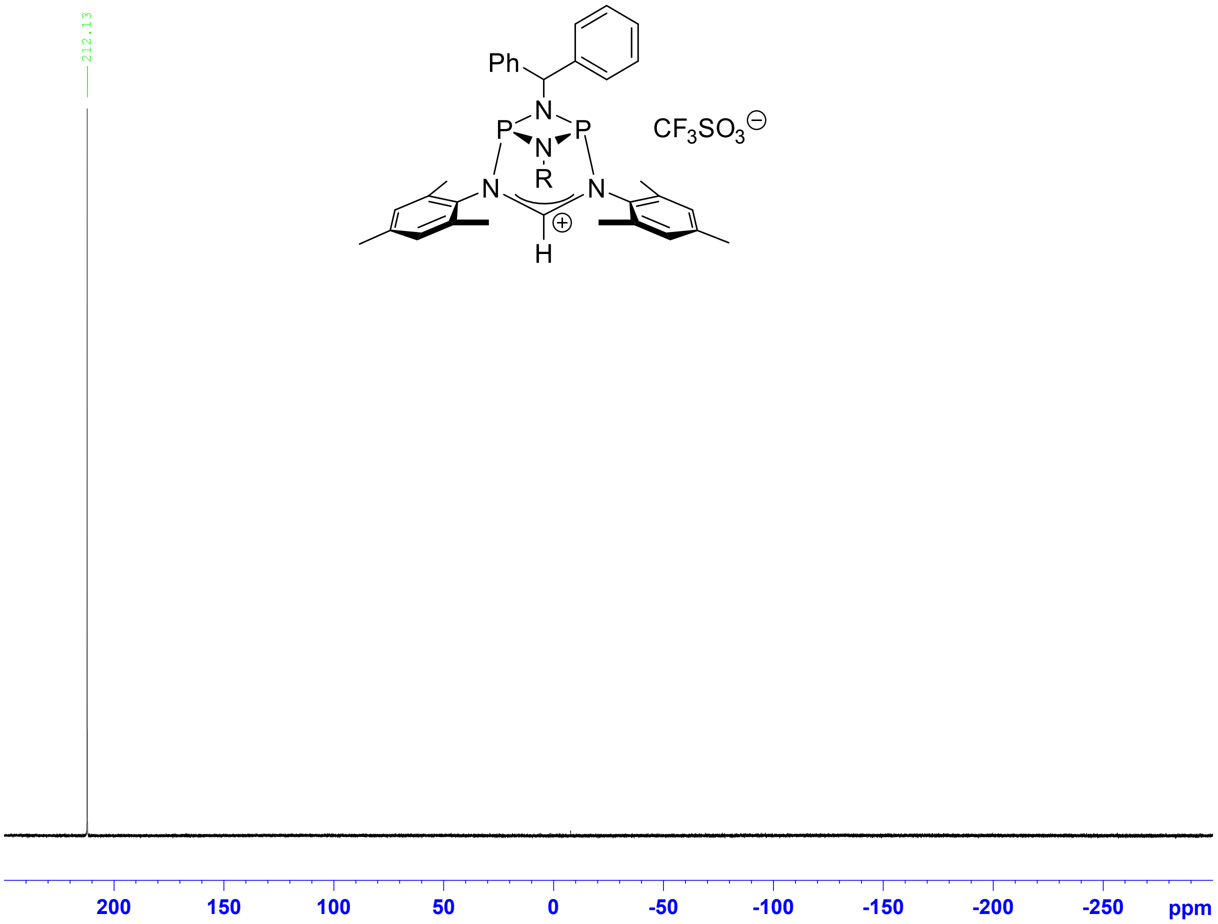

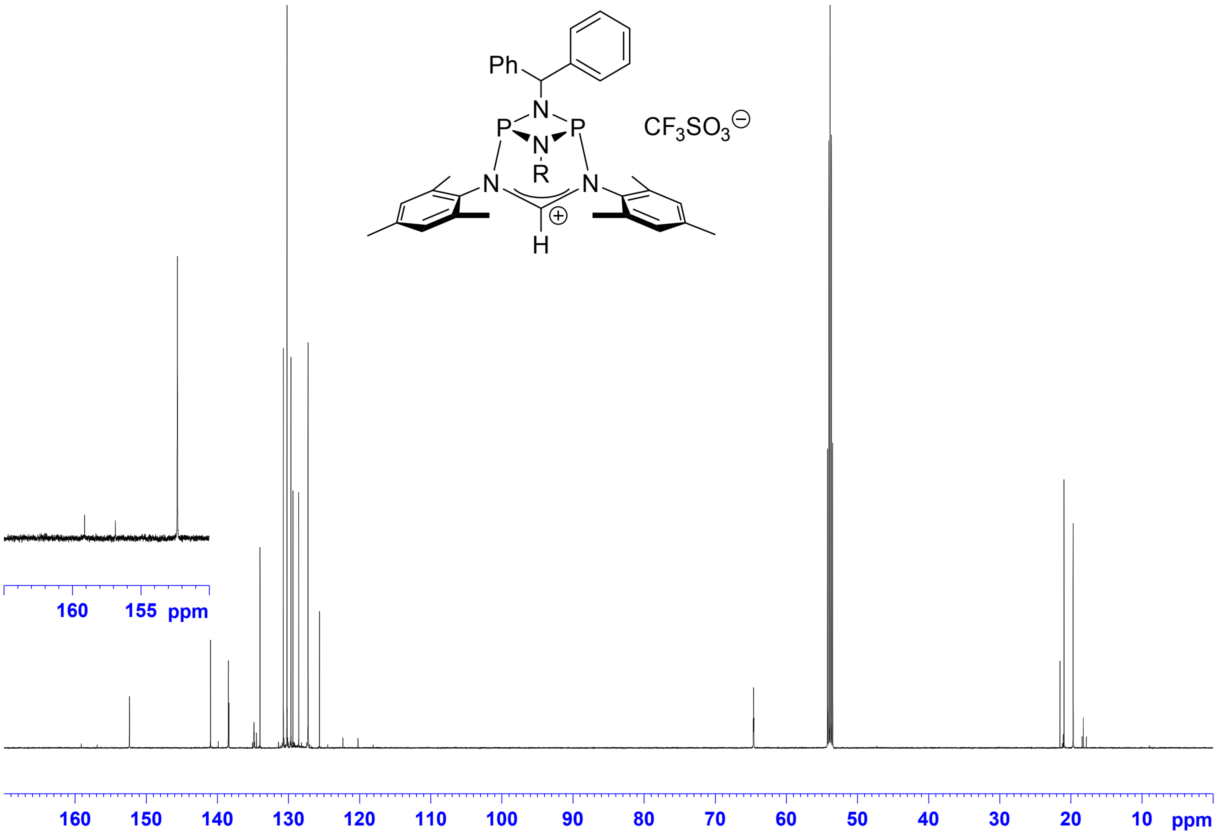

[1]H, ^{31}P{^{1}H} and ^{13}C{^{1}H} NMR spectra of the *in situ* generated carbene **3** in Toluene-d_8. All NMR spectra support a clean deprotonation step, thus no decomposed byproducts are observed.

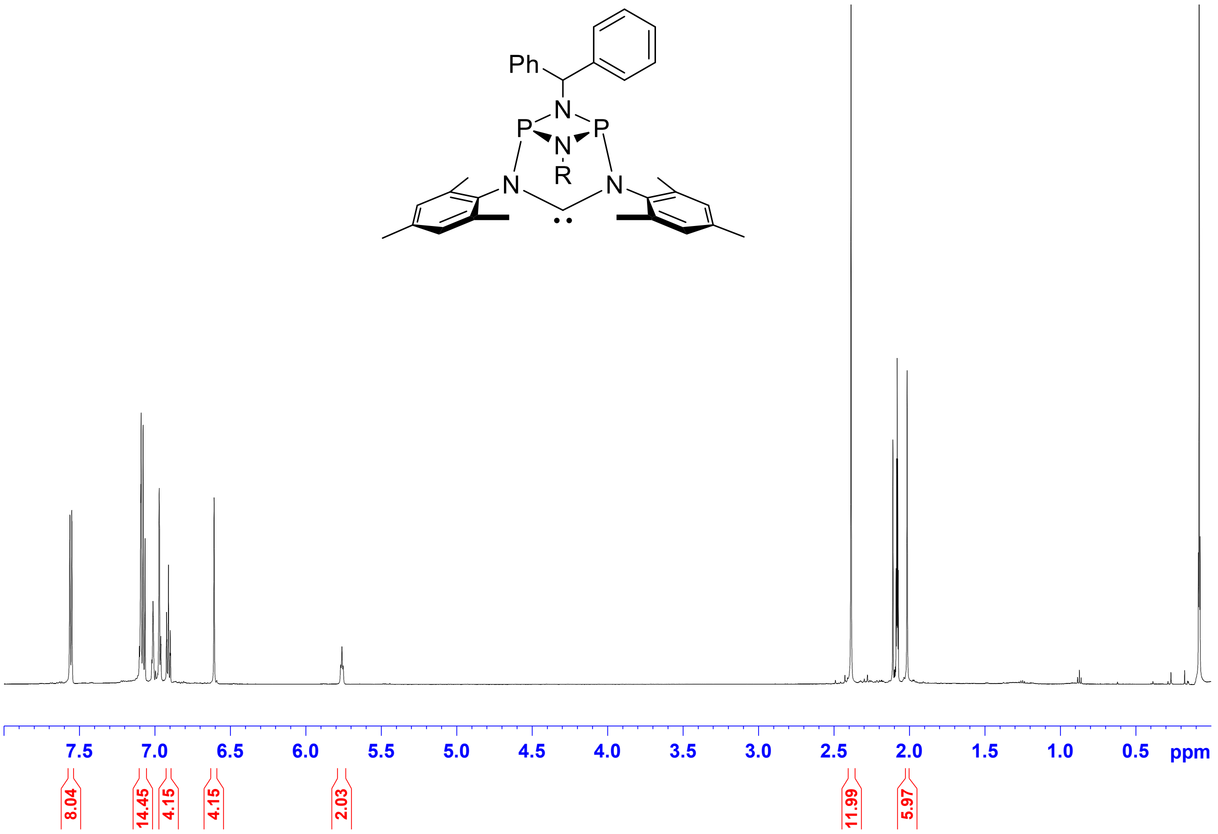

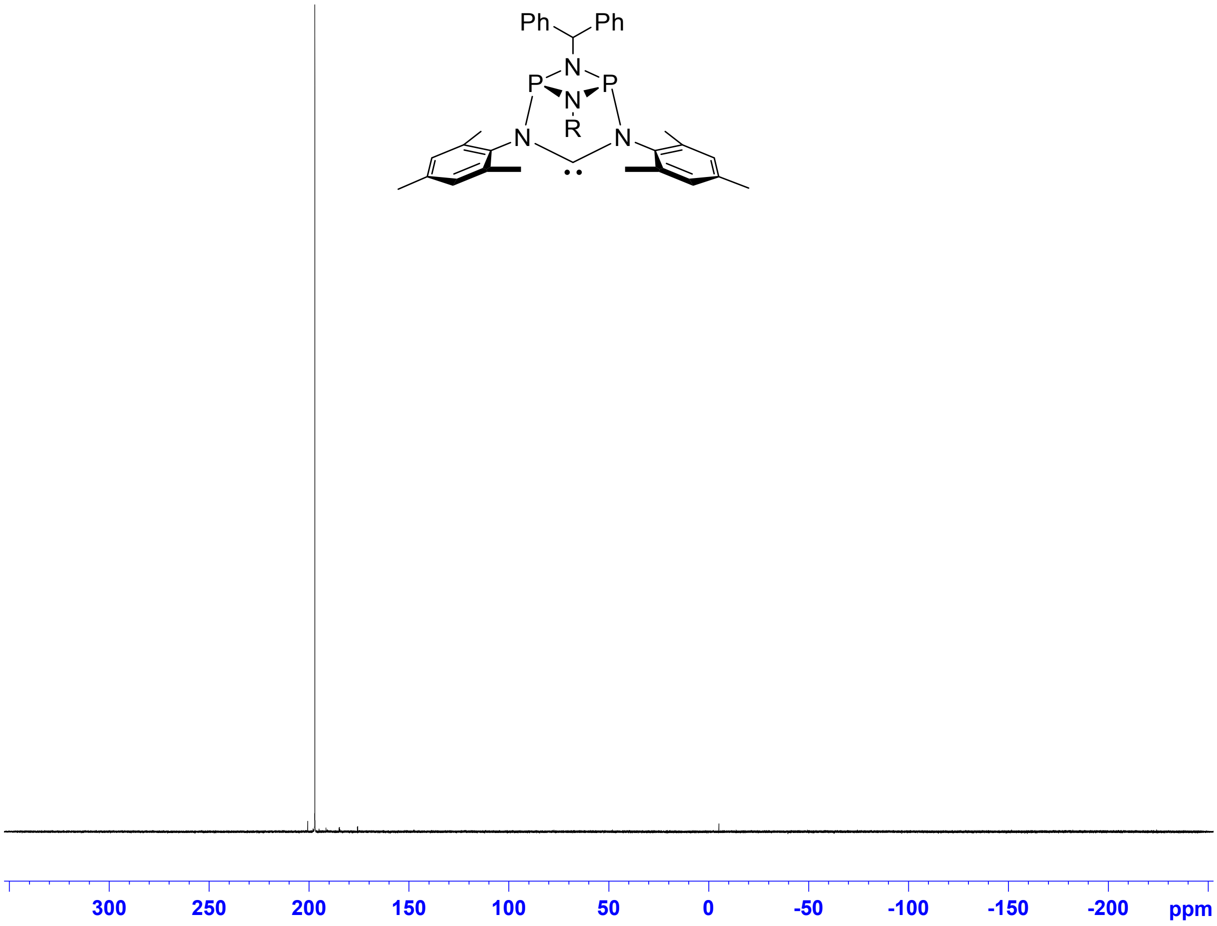
Ph
Ph
N
P
N
P
R
N
N
300
250
200
150
100
50
0
-50
-100
-150
-200
ppm

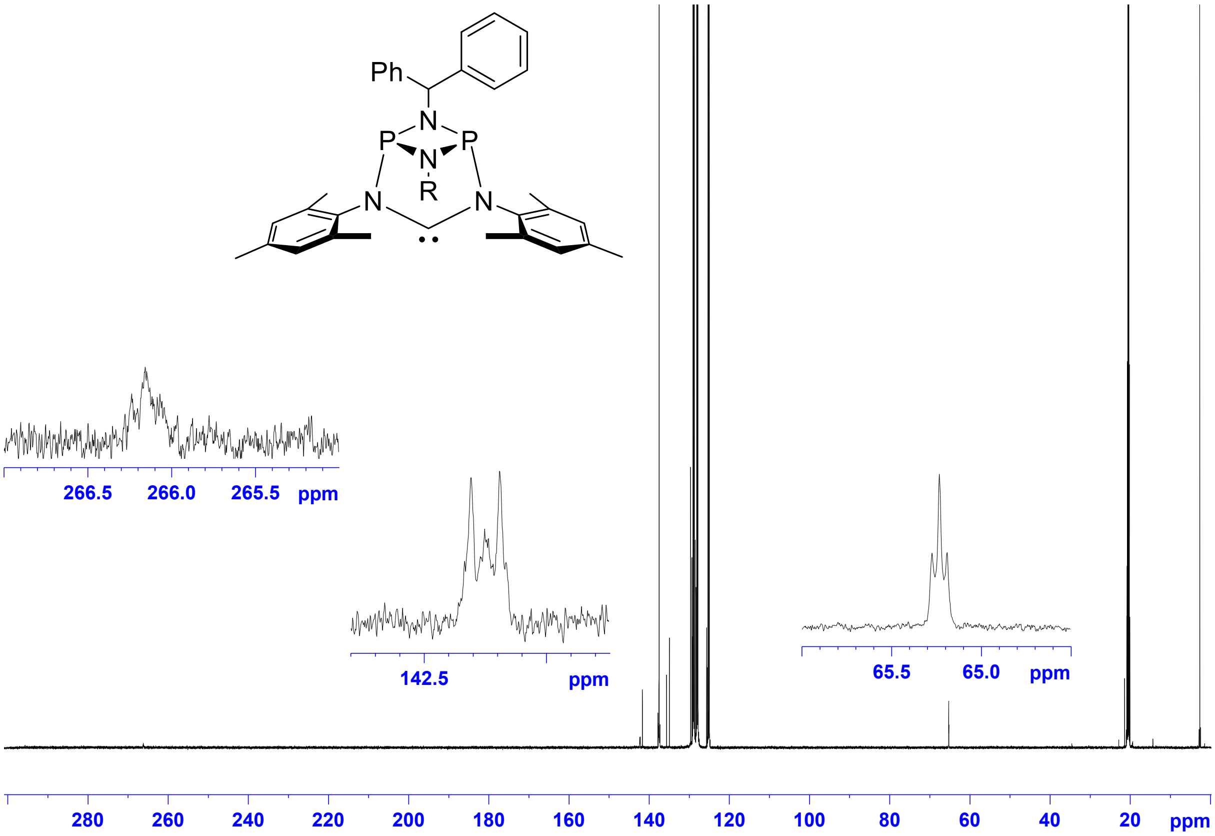
Ph
N
P
N
P
R
N
N
266.5
266.0
265.5
ppm
142.5
ppm
65.5
65.0
ppm
280
260
240
220
200
180
160
140
120
100
80
60
40
20
ppm

^{1}H, $^{31}P\{^{1}H\}$ and $^{13}C\{^{1}H\}$ NMR spectra of single crystals of complex **4** in tetrahydrofuran-d_8.

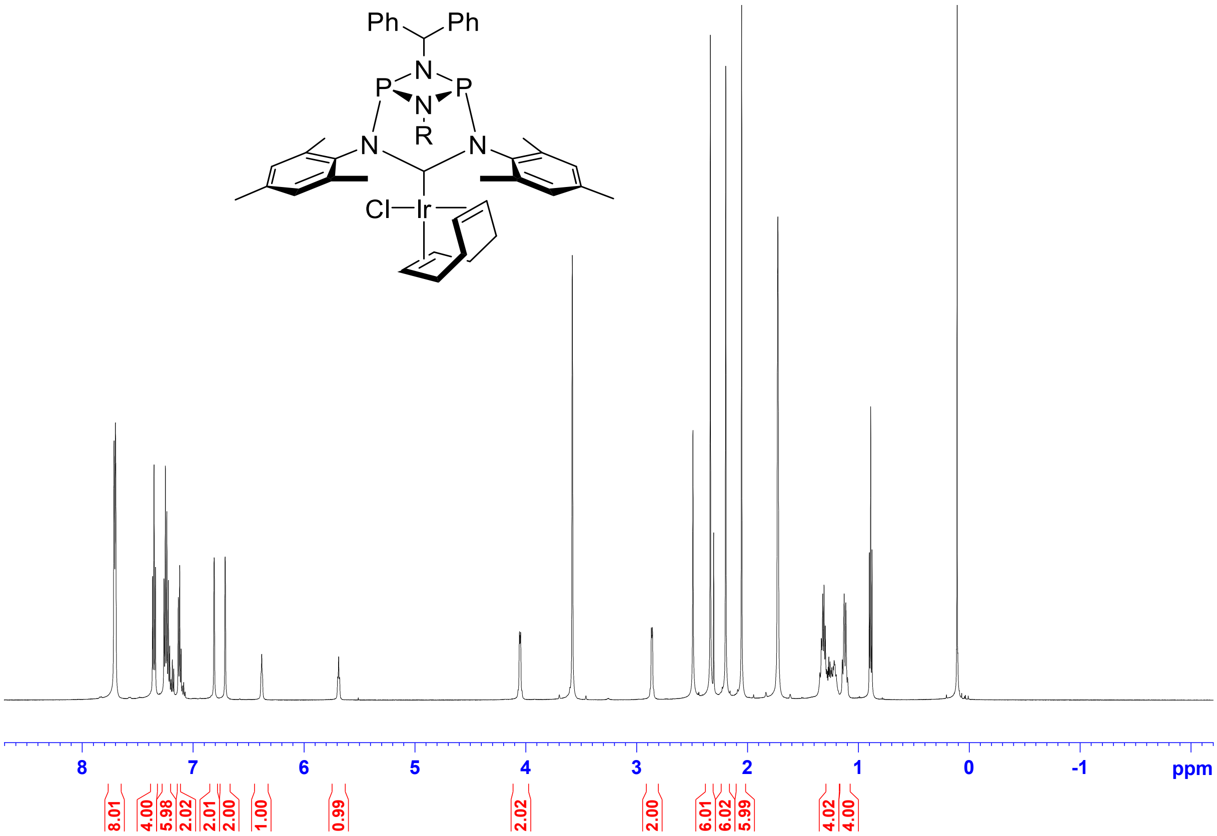

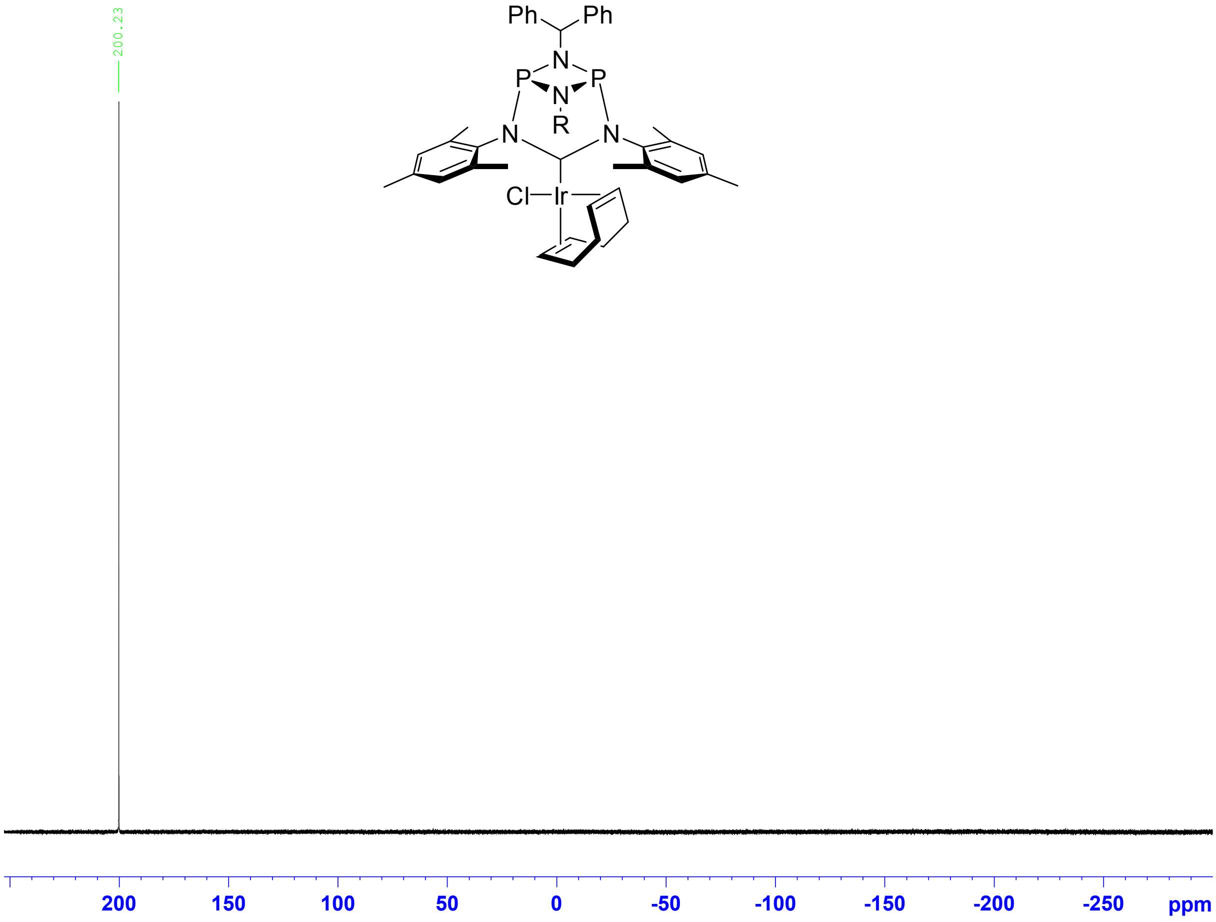

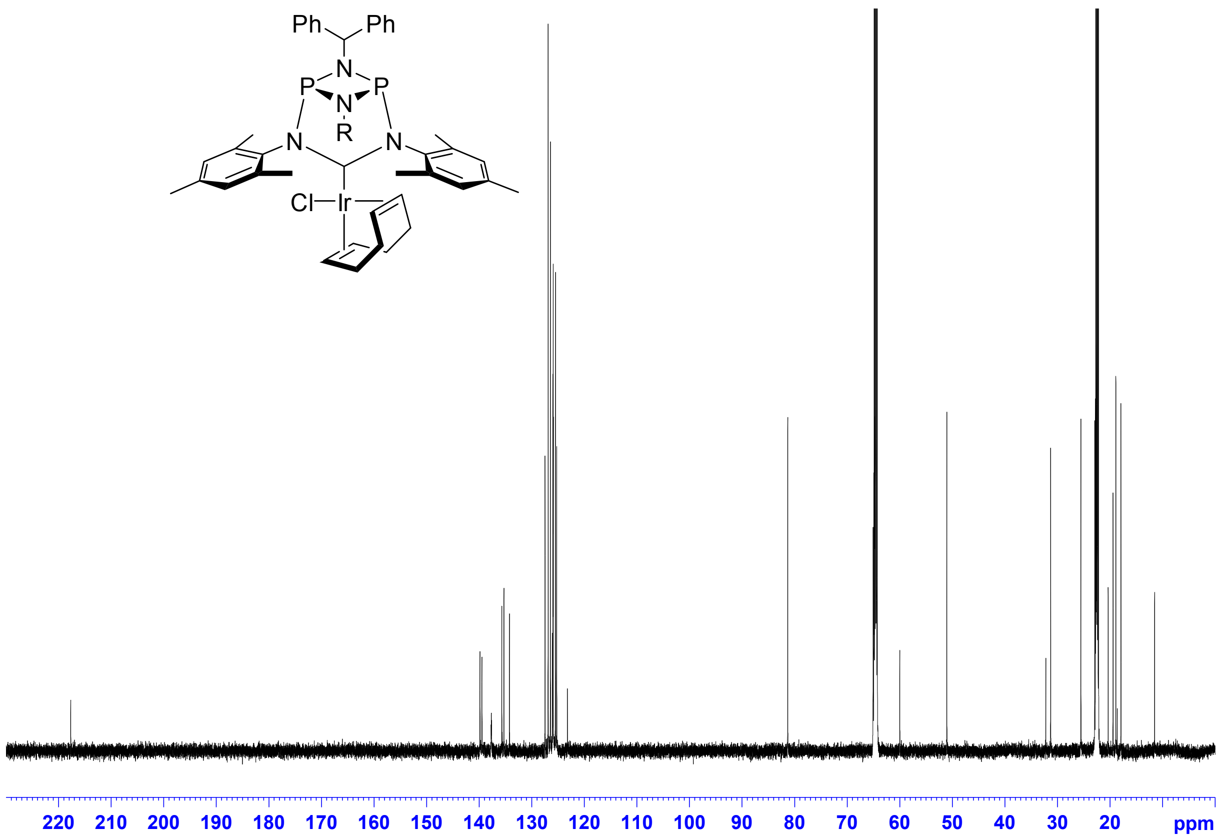

^{1}H, ^{31}P{^{1}H} and ^{13}C{^{1}H} NMR spectra of complex **5** in tetrahydrofuran-d$_8$.

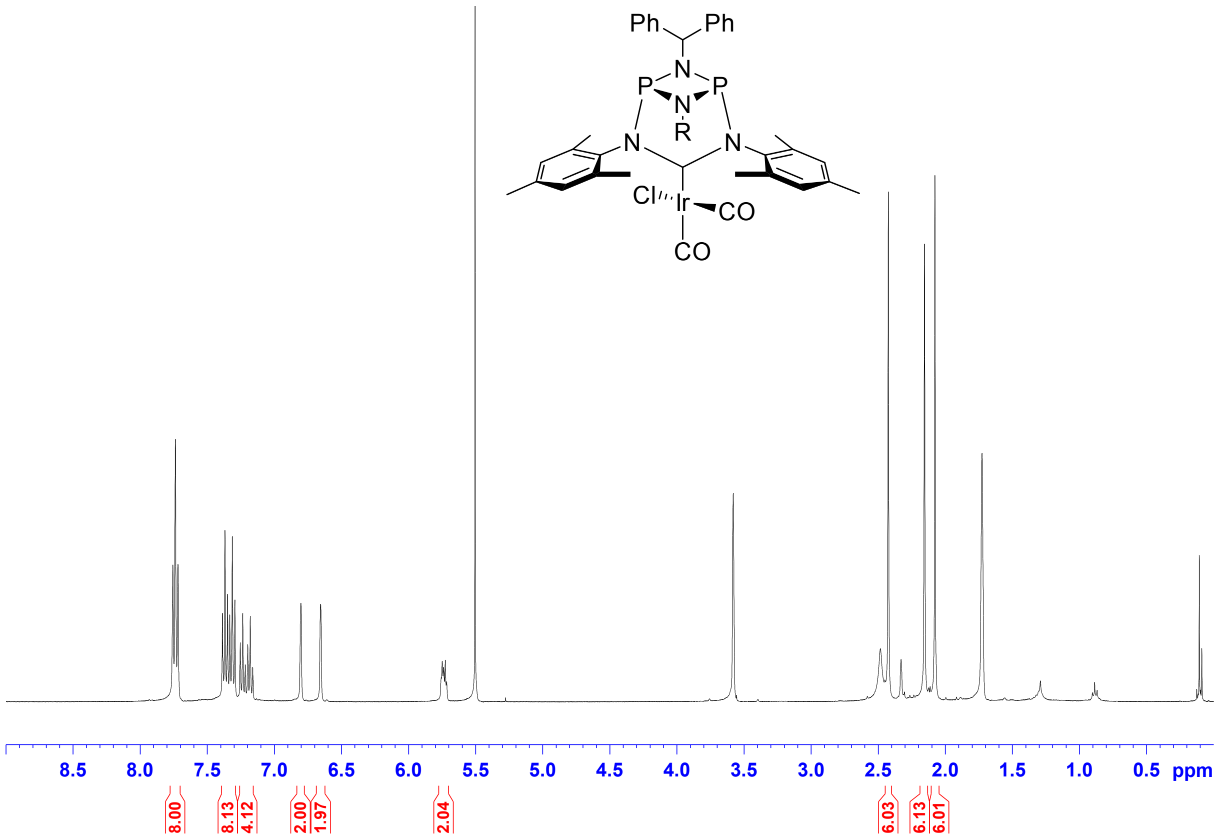

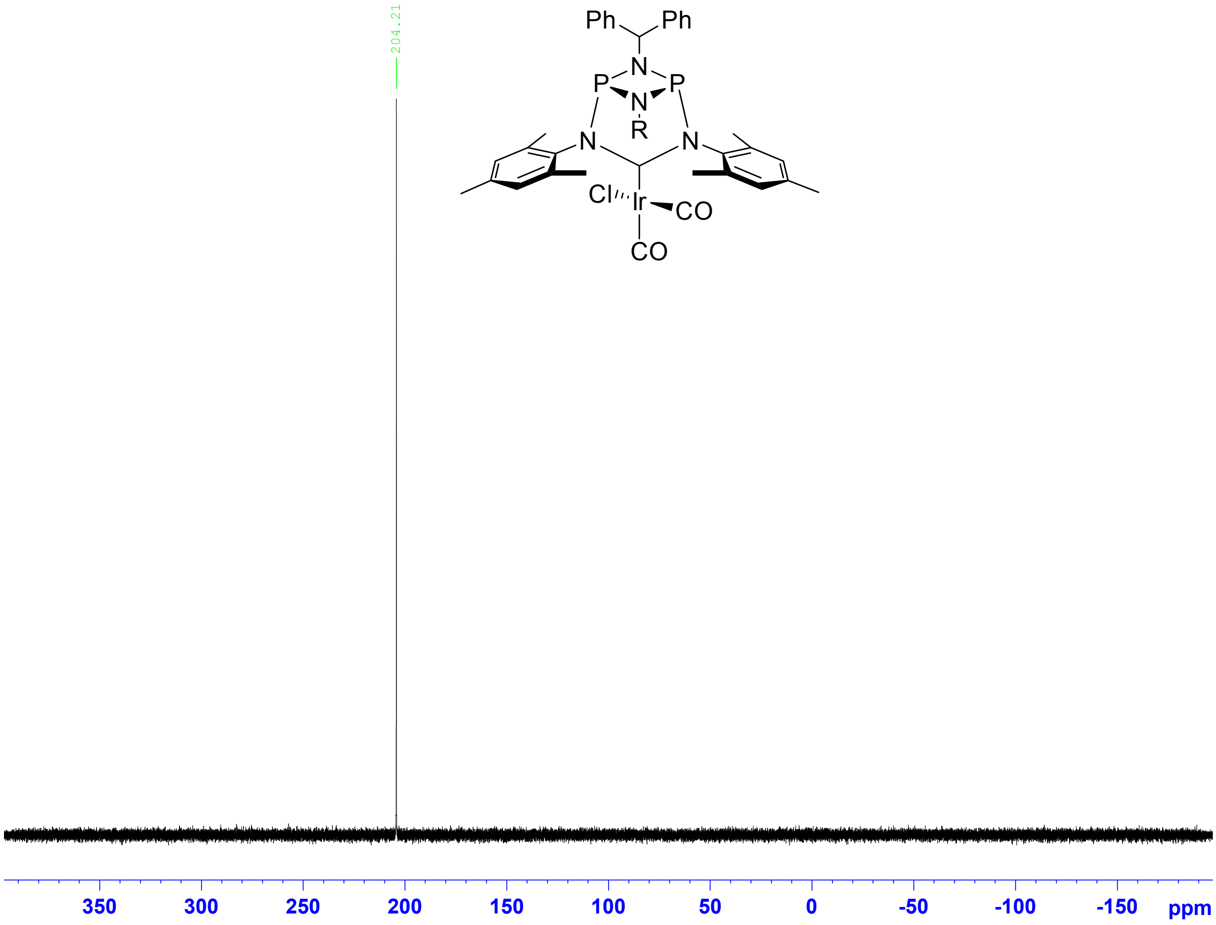
204.21
Ph
Ph
N
P
P
N
R
N
N
Cl
Ir
CO
CO
350
300
250
200
150
100
50
0
-50
-100
-150
ppm

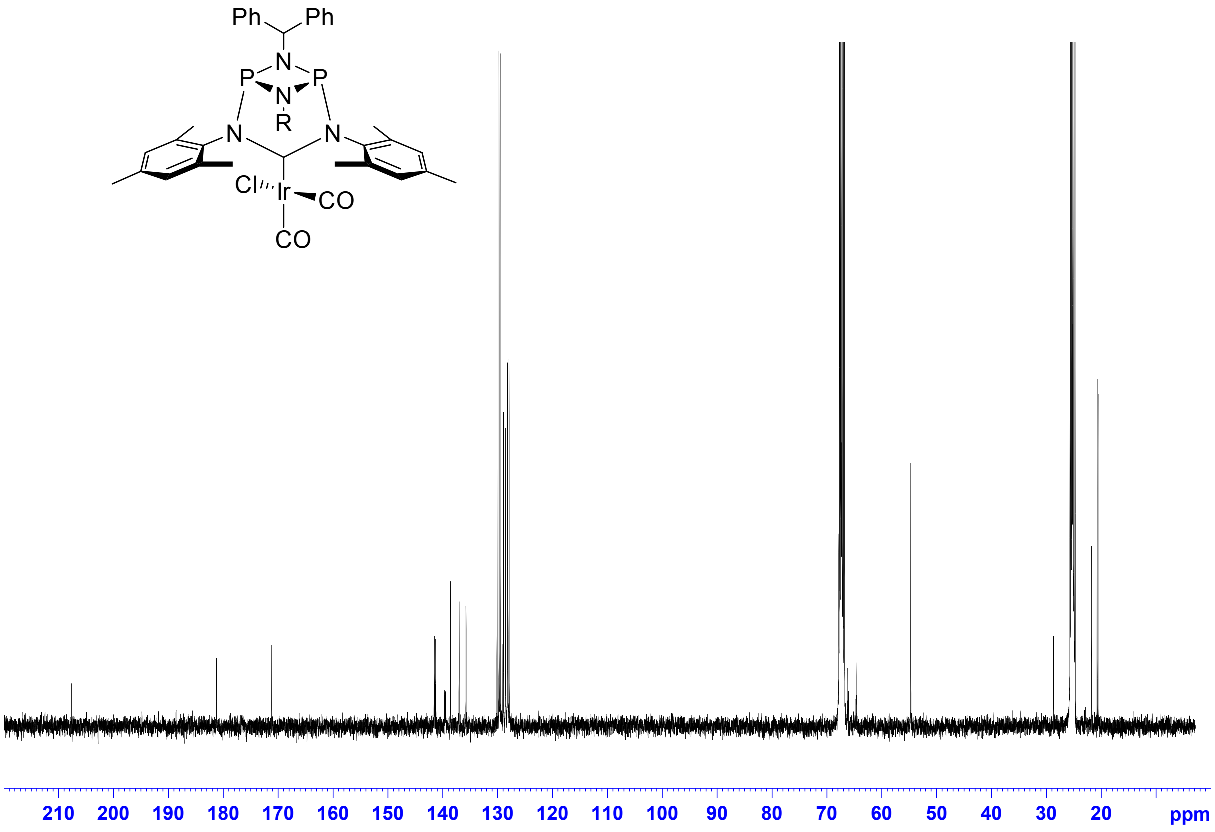
Ph
Ph
N
P
P
N
R
N
N
Cl
Ir
CO
CO
210
200
190
180
170
160
150
140
130
120
110
100
90
80
70
60
50
40
30
20
ppm

^{1}H, $^{31}P\{^{1}H\}$ and $^{13}C\{^{1}H\}$ NMR spectra of single crystals of complex **6** in tetrahydrofuran-d_8.

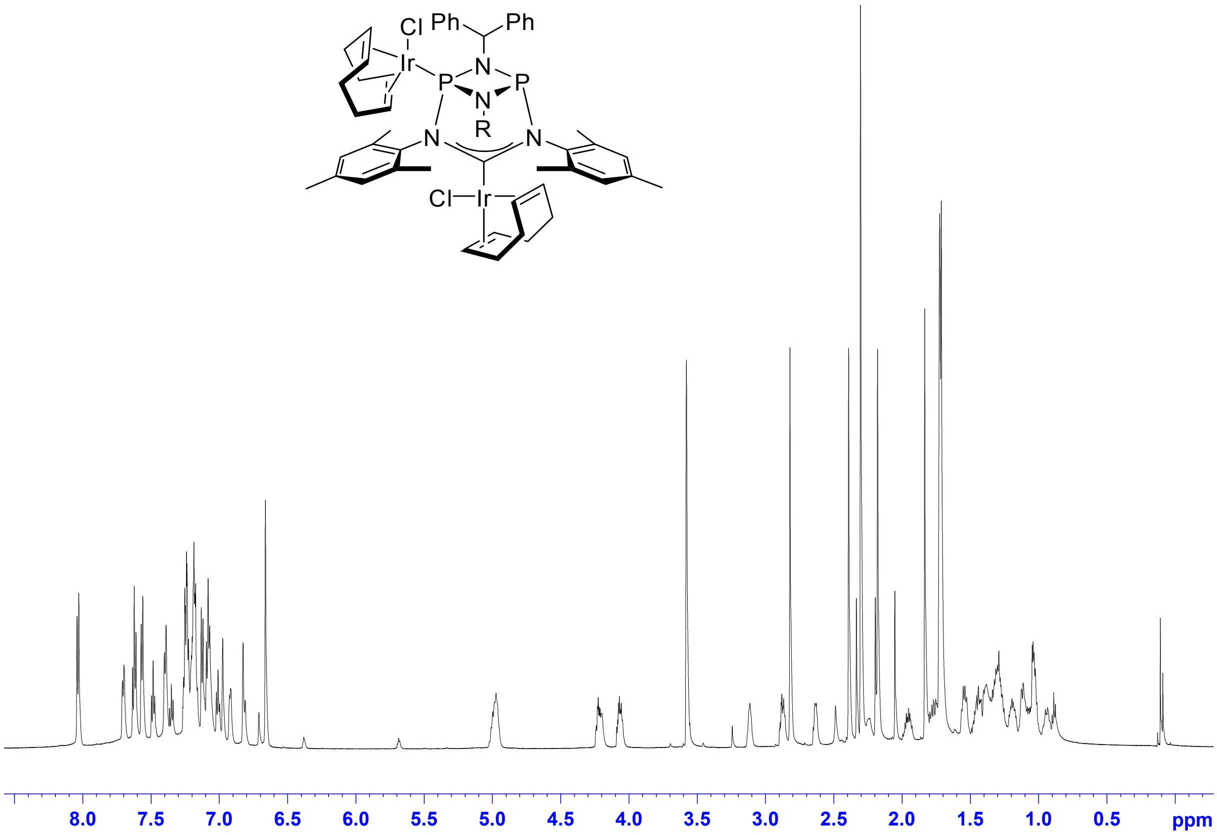

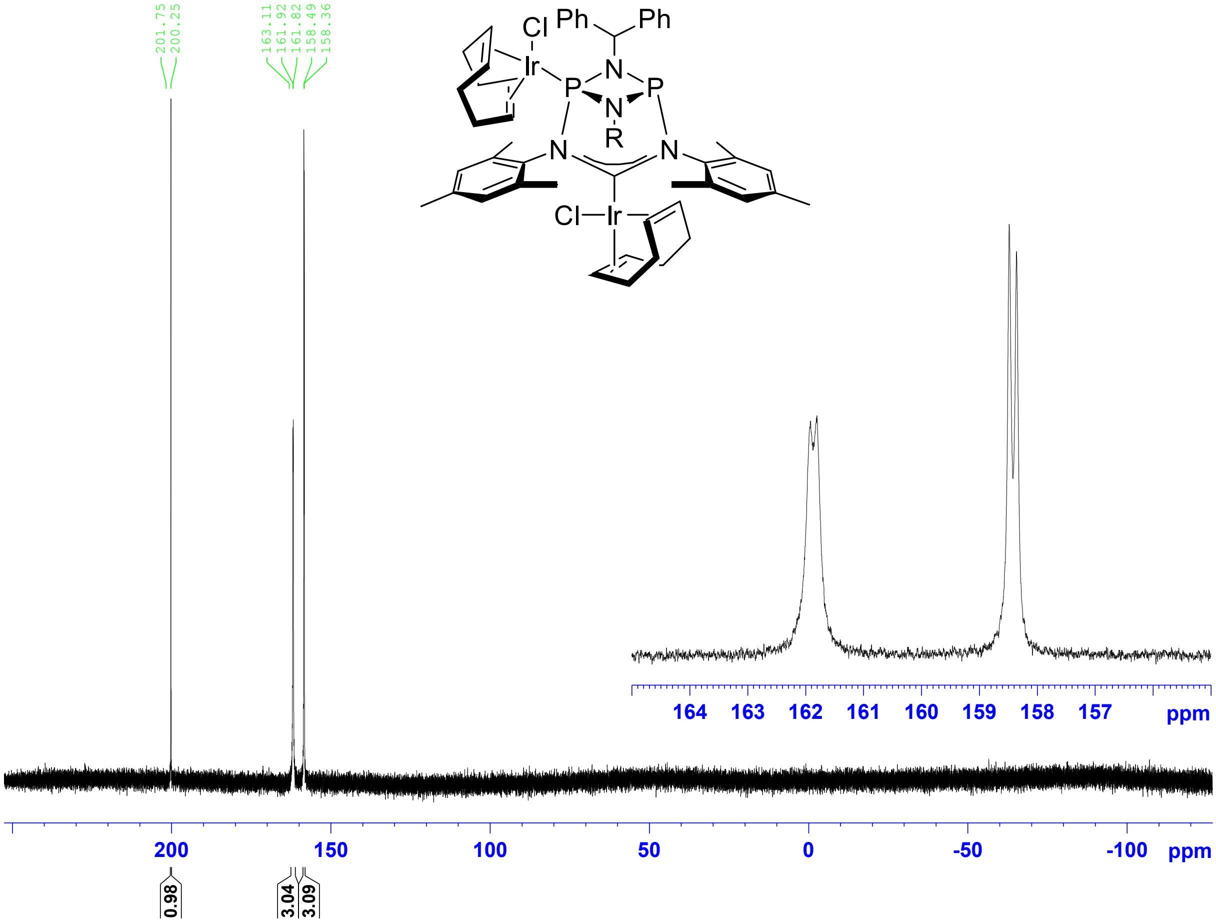

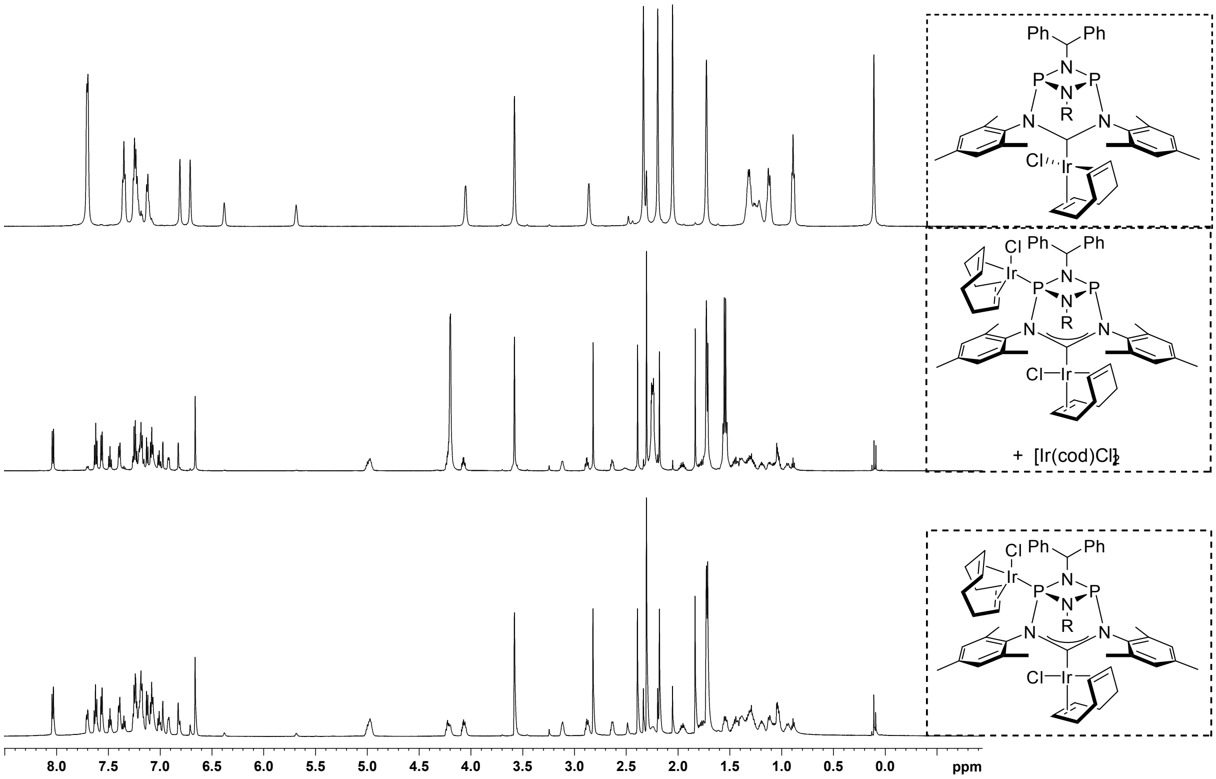

^{1}H, $^{31}P\{^{1}H\}$ and $^{13}C\{^{1}H\}$ NMR spectra of single crystals of complex **7** in tetrahydrofuran-d$_8$.

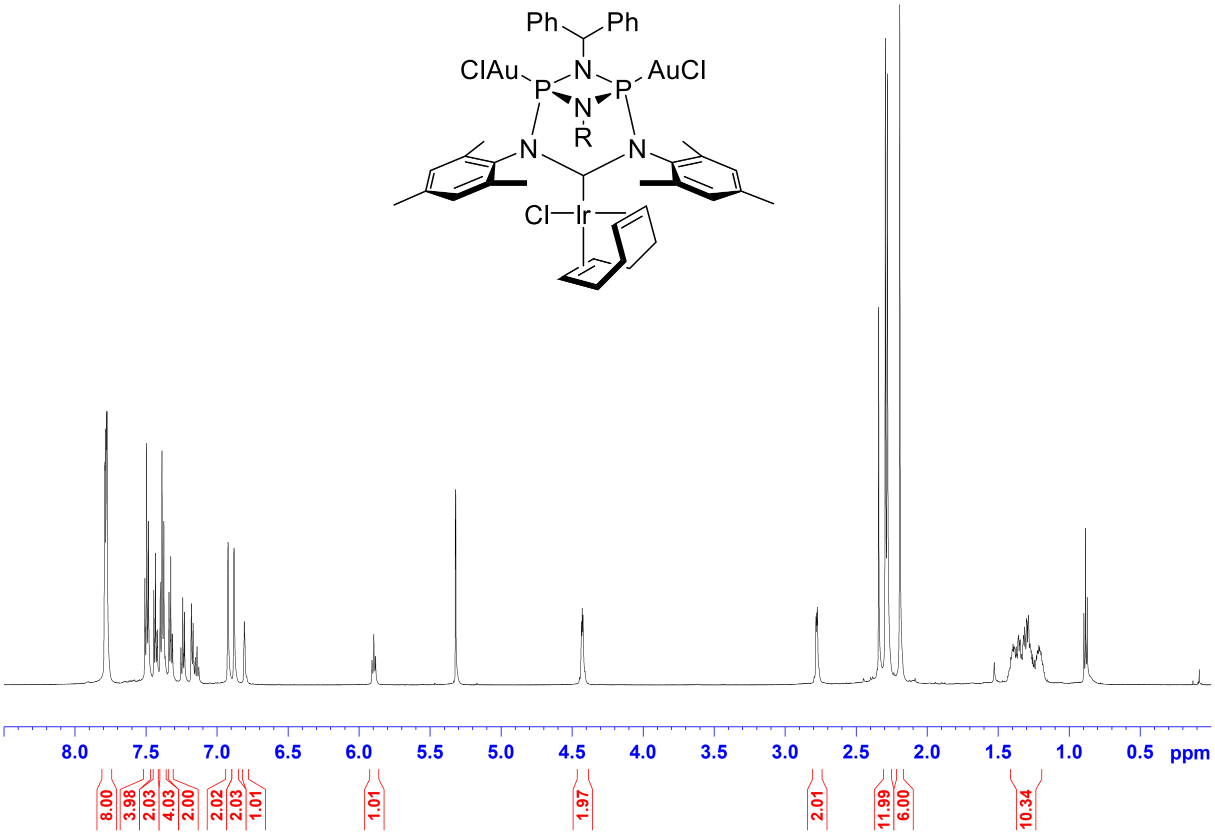

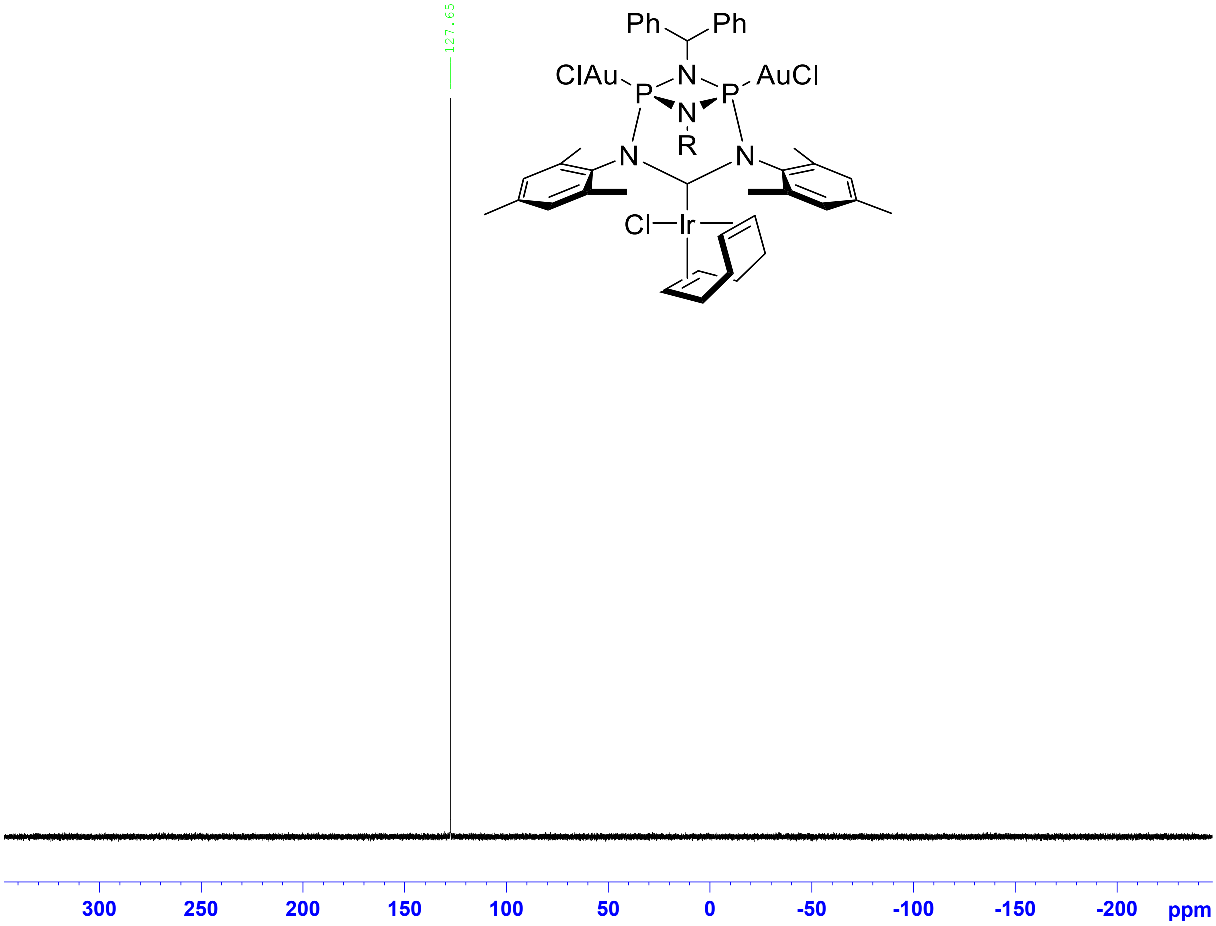
127.65
Ph Ph
ClAu P N P AuCl
N R
N N
Cl Ir
300 250 200 150 100 50 0 -50 -100 -150 -200 ppm

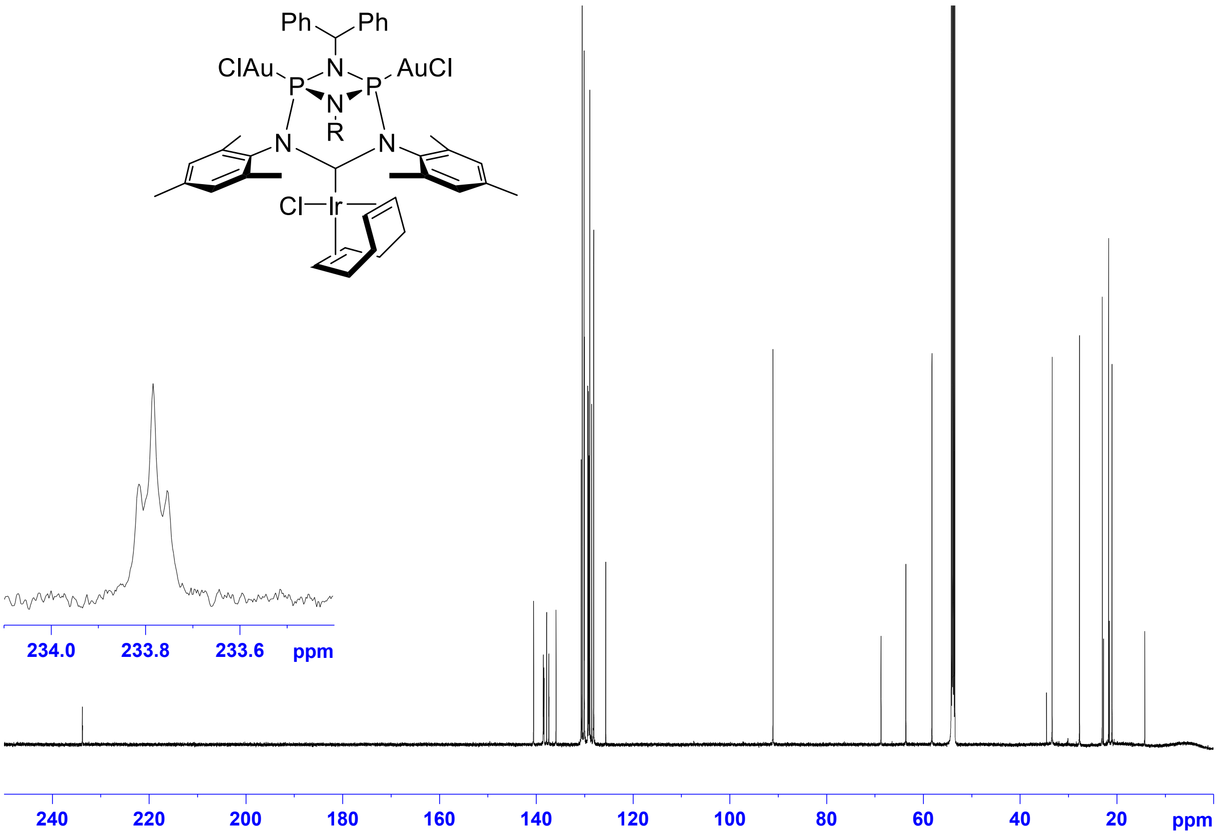
Ph Ph
ClAu P N P AuCl
N R
N N
Cl Ir
234.0 233.8 233.6 ppm
240 220 200 180 160 140 120 100 80 60 40 20 ppm

10. References

[1] W. L. F. Armarego, C. L. L. Chai, *Purification of Laboratory Chemicals*, Butterworth-Heinemann, 7th edition, **2012**.

[2] a) G. R. Fulmer, A. J. M. Miller, N. H. Sherden, H. E. Gottlieb, A. Nudelman, B. M. Stoltz, J. E. Bercaw, K. I. Goldberg, *Organometallics* 2010, **29**, 2176-2179; b) H. E. Gottlieb, V. Kotlyar, A. Nudelman, *J. Org. Chem.* 1997, **62**, 7512-7515.

[3] T. Roth, H. Wadepohl, D. S. Wright, L. H. Gade, *Chem. Eur. J.* 2013, **19**, 13823-13837.

[4] K. M. Kuhn, R. H. Grubbs, *Org. Lett.* 2008, **10**, 2075-2077.

[5] K. Hirano, S. Urban, C. Wang, F. Glorius, *Org. Lett.* 2009, **11**, 1019-1022.

[6] E. Despagnet-Ayoub, R. H. Grubbs, *J. Am. Chem. Soc.* 2004, **126**, 10198-10199.

[7] N. G. Connelly, W. E. Geiger, *Chem. Rev.* 1996, **96**, 877-910.

[8] *CrysAlisPro*, Agilent Technologies UK Ltd., Oxford, UK **2011-2014**.

[9] R. H. Blessing, *Acta Cryst.* 1995, **A51**, 33; *SCALE3 ABSPACK, CrysAlisPro*, Agilent Technologies UK Ltd., Oxford, UK **2011-2014**.

[10] (a) L. Palatinus, *SUPERFLIP*, EPF Lausanne, Switzerland, **2007-2014**; (b) L. Palatinus, G. Chapuis, *J. Appl. Cryst.* 2007, **40**, 786.

[11] (a) G. M. Sheldrick, *SHELXL-20xx*, University of Göttingen and Bruker AXS GmbH, Karlsruhe, Germany **2012-2014**; (b) G. M. Sheldrick, *Acta Cryst.* 2008, **A64**, 112.

[12] P. v. d. Sluis, A. L. Spek, *Acta Cryst.* 1990, **A46**, 194.

[13] (a) A. L. Spek, *PLATON*, Utrecht University, The Netherlands; (b) A. L. Spek, *J. Appl. Cryst*. 2003, **36**, 7.

[14] D. Braga, T. F. Koetzle, *J. Chem. Soc., Chem. Commun.* 1987, 144.

[15] F. H. Allen, *Acta Cryst.*, 2002, **B58**, 380; CSD version 5.35 of May 2014.

[16] (a) A. C. Hillier, W. J. Sommer, B. S. Yong, J. L. Petersen, L. Cavallo, S. P. Nolan, *Organometallics* **2003**, *22*, 4322-4326; (b) H. Clavier, S. P. Nolan, *Chem. Commun.* **2010**, *46*, 841-861.

[17] A. Poater, B. Cosenza, A. Correa, S. Giudice, F. Ragone, V. Scarano, L. Cavallo, *Eur. J. Inorg. Chem.* **2009**, *13*, 1759-1777.
